# Supplementary material for: Effect of a scaled-up quality improvement intervention on health workers’ competence on neonatal resuscitation in simulated settings in public hospitals: A pre-post study in Nepal
Source: PLoS One. 2021 Apr 29;16(4):e0250762. doi: 10.1371/journal.pone.0250762 (PMC8084235; doi:10.1371/journal.pone.0250762)
Supplement: S1 Text — (PDF) [file pone.0250762.s001.pdf]

QUALITY IMPROVEMENT OF PERINATAL CARE

Guideline for Implementation in Hospitals

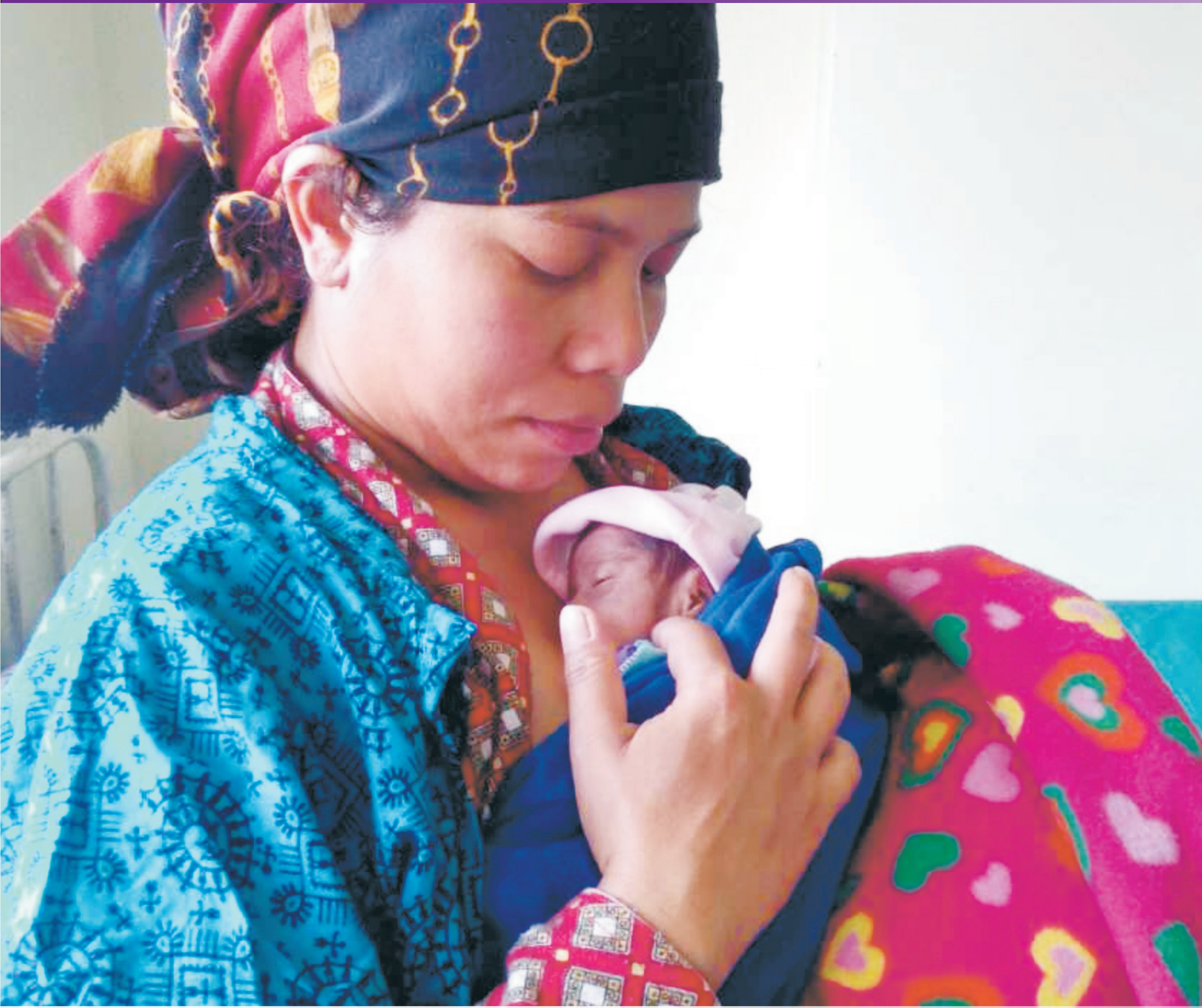

Government of Nepal
Ministry of Health
Department of Health Services
Child Health Division/ Family Health Division
2073

Government of Nepal
Ministry of Health
Department of Health Services

Teku
Kathmandu, Nepal

Date: ....

Preface

It gives me an immense pleasure to know that Child Health Division and Family Health Division have entered into a formal process of improving quality of perinatal care services through the development of this guideline. Newborn health and stillbirth remain a major challenge in developing countries like Nepal. Since a large proportion of under-five mortality is still occupied by newborn deaths, increasing investments for improving the survival of newborns through universal access to evidence-based intervention is vital. Moreover, without improvement in overall quality of services, further improvements cannot be expected. Improving the quality of care in health facilities is increasingly recognized as an important attention in the course of ending preventable mortality and morbidity among newborns.

I would like to thank Child Health Division, Family Health Division and all the stakeholders who are involved in developing this guideline. I hope it will prove itself to be an important milestone for further improvement in childhood survival in Nepal.

I would like to call upon all the partners, managers and health service providers for the effective implementation of this guideline; Quality Improvement of Perinatal Care to achieve desired outcomes in perinatal health.

Director General

Dr. Rajendra Pant
Director General,
Department of Health Services

Government of Nepal
Ministry of Health
Department of Health and Services
Child Health Division

Director: 46261660
EPI: 4262263
IMNCI: 4219218
Nutrition :4225558
P&M: 42666573
Fax: 4262238
Email: chd@mohp.gov.np
childhealthimci@gmail.com
website: www.chd.gov.np

Government of Nepal
Ministry of Health
Department of Health Services
Teku, Kathmandu

Ref.No: ३६३ (363)

Way Forward

With the remarkable progress in child survival, Nepal has been able to achieve Millennium Development Goals (MDGs) of reducing under-five mortality in the country. However, neonatal health and stillbirths represent the part of unfinished agenda of MDGs. Acceleration in reduction of neonatal mortalities calls for extra efforts in overcoming existing barriers. One of the barriers in improved neonatal health outcome is quality of care provided during the perinatal period. To end preventable newborn morbidity and mortality, every newborn should have skilled care at birth with evidence-based practices delivered in a humane, respectful, supportive environment. The outcome of care for women and newborns around the time of birth in health facilities reflects the evidence-based practices used and the overall quality of services provided.

Child Health Division has taken this initiative to improve the availability and quality of health services for newborns in hospital settings. This guideline has been developed to provide direction to managers and service providers to set up an enabling environment, identify gaps in service quality and to establish sustainable mechanism for quality perinatal care services.

I would like to thank **Mr. Parashu Ram Shrestha**, IMNCI section chief, for taking this initiation. I am thankful to UNICEF Nepal for providing technical assistance in developing this concept of *"Quality Improvement of Perinatal Care"*. I would also like to thank all the partners, academia and professional bodies for their valuable inputs in the course of developing this guideline.

Lastly, I heartily request our partners to extend their support to improve quality of perinatal care for better outcomes in newborn survival.

....

Dr. Bikash Lamichhane
Director,
Child Health Division

List of Abbreviations

**AIDS**	Acquired Immune Deficiency Syndrome
**CHD**	Child Health Division
**D(P)HO**	District (Public) Health Office
**ENAP**	Every Newborn Action Plan
**HBB**	Helping Babies Breathe
**HIV**	Human Immune Deficiency Virus
**IMNCI**	Integrated Management of Neonatal and Childhood Illness
**MDG**	Millennium Development Goals
**MPDR**	Maternal and Perinatal Death Review
**NeNAP**	Nepal's Every Newborn Action Plan
**NHTC**	National Health Training Center
**NICU**	Newborn Intensive Care Unit
**OPD**	Out Patient Department
**PDSA**	Plan-Do-Study-Act
**QI**	Quality Improvement
**RHD**	Regional Health Directorate
**RHTC**	Regional Health Training Center
**SNCU**	Special Newborn Care Unit
**UNICEF**	United Nations Children's Fund
**WHO**	World Health Organization

Table of Contents

LIST OF ABBREVIATION.

1. BACKGROUND…………………………………………..………............... - 7 -

2. RATIONALE……………………………………………………………............... - 8 -

3. PURPOSE OF THE GUIDELINE…………………………………………....... - 9 -

4. PRIMARY USER OF THE DOCUMENT……………………………………... - 9 -

5. WHY QI OF PERINATAL CARE ?... - 9 -

6. WHAT IS QI OF PERINATAL CARE ?.. - 9 -

7. FUNDAMENTALS FOR QI………………………………………………......... - 11 -

  7.1. STRUCTURE FOR IMPLEMENTATION OF QI PROCESS…………… - 11 -

  7.2. STANDARDS FOR PERINATAL CARE……………………………..…. - 11 -

  7.3. PROCESS/SYSTEM……………………………………….............. - 12 -

  7.4. MEASUREMENT OF CHANGE……………………………………........ - 12 -

8. IMPLEMENTATION STRATEGIES……………………………………......... - 14 -

9. IMPLEMENTATION APPROACH…………………………………………...... - 14 -

9.1. PREPARATORY PHASE ……………………….……………….......... - 15 -

- *SELECTION AND ORIENTATION OF MENTORS………………………..* - 15 -
- *ORIENTATION OF QI/MPDR COMMITTEE ON PERINATAL QI PACKAGE..* - 15 -
- *SELECTION OF QI FACILITATORS……..…………………………………* - 16 -
- *TRAINING OF MENTORS AND QI FACILITATORS………………………* - 17 -
- *ORIENTATION TO UNIT STAFFS ON PERINATAL QI PACKAGE………* - 18 -
- *ASSESSMENT OF THE READINESS, AVAILABILITY AND QUALITY OF PERINATAL CARE SERVICE……………………………………………* - 18 -
- *CONDUCT CAUSAL/BOTTLENECK ANALYSIS IN PROVIDING QUALITY PERINATAL SERVICES……...……………………...................* - 20 -
- *DEVELOPMENT OF ON-SITE PLAN TO IMPROVE THE QUALITY OF PERINATAL CARE………...……………………………………………........* - 21 -

- *MOBILIZATION OF RESOURCES FOR AVAILABILITY OF PERINATAL CARE EQUIPMENT*………………………………………………………….. - 22 -
- *SET UP ROUTINE SYSTEM TO MONITOR THE PROGRESS IN CARE FOR SICK NEWBORN*……………………………………………………...... - 23 -

9.2. IMPLEMENTATION PHASES…………………………………………........ - 24 -

- *CAPACITY BUILDING OF HEALTH WORKERS ON WHO'S/ NATIONAL NEWBORN CLINICAL STANDARDS AND QI IMPLEMENTATION PROCESS*…... - 24 -
- *PROVISION OF QI TOOLS*……………………………………………....... - 25-
- *IMPLEMENTATION OF PDSA CYCLE TO IMPROVE QUALITY OF CARE*…….. - 25 -
- *UNIT MEETINGS (PDSA MEETINGS)*………………………………......... - 26 -
- *REFRESHER TRAINING TO HEALTH WORKERS*……………………….. - 26 -

9.3. SUSTAINING THE CHANGE…………………………………………........ - 27 -

- *CONTINUOUS ASSESSMENT OF THE SERVICE READINESS, AVAILABILITY AND QUALITY OF PERINATAL CARE*……….................. - 27-

ANNEXES……………………………………………………………….................. - 29 -

1. Background

Newborn Health, a part of unfinished agenda of Millennium Development Goals, calls for concentrated and evidence based interventions in place with good quality. There has been remarkable progress in reducing the number of child deaths globally and in Nepal, in recent decades. However, 2.9 million babies die every year within the first month of life and an additional 2.6 million babies are stillborn globally. In 2010, of the total 2.65 million third trimester stillbirths that occur every year, more than half (1.45 million) occurred during the antepartum period and the remaining during the intra-partum period. In this context, the global Every Newborn Action Plan (ENAP) has defined priority actions to address preventable causes of neonatal mortality i.e. preterm birth complication, intra-partum related complications, and infections.

Nepal has achieved the MDG 4 target where under-five mortality rate fell from 142 deaths per 1,000 live births in 1990 to 38 in 2015. In 2015, neonatal death occupied 61 percent of under-five deaths making it clear that further decline in the overall under-five mortality rate is not possible without significant reduction of neonatal deaths. Out of the total 12975 newborn deaths in 2013, the primary cause was preterm birth complications (31%), followed by intra-partum related events (birth asphyxia or trauma, 23%) and newborn infection (excluding pneumonia or acute lower respiratory infections and HIV/AIDS, including sepsis, tetanus, pertussis and other newborn infections, 19%).

Nepal's Newborn Action Plan (NeNAP) has pledged to reduce neonatal mortality rate to 11 per 1000 live births and stillbirth rate to 13 per 1000 total births by 2030. The period around childbirth is the most critical for saving the maximum number of maternal and newborn lives and preventing stillbirths. This is because, with increasing numbers of births in health facilities, more avoidable maternal and perinatal mortality and morbidity are occurring in those facilities. In 2014, 54 percent of all deliveries occurred in health institutions in Nepal, which is more than a fourfold increase from 2001. Given the context of increase in institutional deliveries in hospitals, improving quality of care at birth and for high-risk newborn is important to reduce in-hospital stillbirth and neonatal death. The targets of reducing maternal, neonatal deaths and stillbirths will not be achieved without improving the quality of care around the time of birth and for small and sick newborns.

To end preventable maternal and newborn morbidity and mortality, every pregnant woman and newborn should have skilled care at birth with evidence-based practices delivered in a humane, respectful, supportive environment. Good quality care requires appropriate use of effective clinical and non-clinical interventions, strengthened health infrastructure, optimum skills and a positive attitude of health providers. These will improve health outcomes and give women, their families and the health care providers a positive experience. High-quality care is integral to the right to health and the route to equity and the preservation of dignity for women and children.

2. Rationale

The efforts made during the period have substantially improved the number of births in health facilities, the proportion of deliveries attended by skilled health personnel in developing countries having increased from 56% in 1990 to 68% in 2012. However, reductions in maternal and neonatal mortality remain slow. With increasing numbers of births in health facilities, attention has shifted to the quality of care, as poor quality of care during pregnancy, childbirth and in the postnatal period significantly contributes to the annual estimated 289,000 maternal deaths, 2.6 million stillbirths and 2.8 million newborn deaths globally.

The outcome of the care for women and newborns around the time of birth in health facilities reflects the evidence-based practices used and the overall quality of services provided. The quality of care depends on the physical infrastructure, human resources, knowledge, skills and capacity to deal with both normal pregnancies and complications that require prompt, life-saving interventions. Improving the quality of care in health facilities is thus increasingly recognized as an important focus in the quest to end preventable mortality and morbidity among mothers and newborns.

An increased focus on quality of care at the time of birth has quadruple returns on investment through the reduction of maternal and neonatal deaths, prevention of stillbirths and future disability. Recent estimates indicate that closure of the quality gap through the provision of effective care for all women and newborn babies delivering in facilities could prevent an estimated 113,000 maternal deaths, 531,000 still births, and 1.32 million neonatal deaths annually by 2020.

Improving the quality of care around birth will save lives and requires functional health system. The issue of quality of care remains central to maternal and newborn health since increasing coverage of interventions alone will not necessarily deliver the outcomes or impact needed to reach mortality reduction targets.

Ending preventable maternal and newborn deaths and stillbirths will be one of the major focuses of Ministry of Health in the coming years, through the commitment made in NeNAP. These targets will not be achieved without improving the quality of care around the time of birth and for small and sick newborns.

In this regard, Department of Health Services, Child Health Division has taken initiative to introduce a multi-faceted 'Quality Improvement (QI)' intervention in hospitals focusing on the improvement of perinatal care quality. The approach is based on WHO's *"Standards for Improving Quality of Maternal and Newborn Care in Health Facilities"* and "*Nepal's Every Newborn Action Plan*". The proposed quality improvement intervention will address disparities in quality of major evidence based services to newborn survival; neonatal resuscitation, kangaroo mother care, breast-feeding and infection prevention and management. The implementation strategies for quality improvement build on previous experiences from Nepal and other similar contexts. Through this initiative, Department of Health Services seeks to establish a concentrated, systematic and targeted approach to strengthen quality of perinatal care with a focus on the context and intervention specific bottlenecks. The quality improvement intervention will thus contribute to reduced perinatal morbidity and mortality by addressing major gaps in quality of newborn care.

3. Purpose of this guideline

The purpose of this guideline is to support program managers on implementation of a QI intervention in hospitals for perinatal care building upon previous experiences from Nepal and similar contexts.

4. Primary user of the document

The primary users of the document are hospital directors, pediatricians, matron, nursing in-charge, Medical Record Officer/medical recorder and health workers working in the delivery unit and sick newborn care unit (Newborn Intensive Care Unit(NICU)/ Special Newborn Care Unit (SNCU). The guideline will also be used by program managers and implementing partners at central, regional and district level to ensure effective implementation of quality improvement processes in hospitals.

5. Why QI of perinatal care

Provision of quality care services most often requires collaborative efforts by health care providers with a range of different skills. QI of perinatal care is based upon the principle that improvement in the quality of clinical care thus requires a multi-disciplinary approach. Improvements take place in organizations when different stakeholders come together to develop a shared understanding of what could be improved, how they–as individuals and teams–can contribute to achieve a common goal, how they can overcome the challenges they foresee or face when aiming to change. Often, the existing communication channels between authorities, leaders of hospitals and health care providers are not adequate for change to come about as a consequence of changes in policy or issues identified in the provision of health services. Based on the previous experience of perinatal care it is therefore imperative to establish a forum to bring together different stakeholders, such as hospital managers, administrators, pediatricians, nurse in-charge, data managers and health workers, to discuss what could be improved, make priorities on which changes to focus on and jointly develop an actionable plan. Such multi-disciplinary teams, where members acknowledge each other's roles and expertise, can function to improve the quality of care based on their joint understanding of current shortcomings in the service readiness, availability and quality of care.

6. What is QI of perinatal care

QI of perinatal care is an effort to institutionalize a quality improvement approach where individuals and teams representing different roles and disciplines jointly take leadership for change management and contribute to improvements aimed towards better health and survival during the perinatal period.

The QI of perinatal care builds upon strengthening the communication between the identified stakeholders and establishing agreement between them with regards to:

- Why is there a need for change?
- What needs to be changed?
- How can that change happen?

   a. Who needs to be involved for change to happen?
   b. What needs to be there to make the change happen?

- How can changes be evaluated and strategies refined?
- How can changes be sustained in the given context?

The above questions call for the need to establish groups that systematically work towards improvement. One method much used globally and previously found to lead to improvements in Nepal is facilitated Plan-Do-Act-Study (PDSA) cycles (see figure 1). The PDSA cycle is a structured approach, recommended by WHO, aiming to identify and act upon locally identified problems. To support the work of multi-professional groups it is common to have a trained facilitator who guides the process and enables an environment in which everyone's voices are heard. To 'facilitate' has been described as a technique by which one person (the facilitator) makes things easier for others (a group of people). To make easier it can be interpreted as supporting, helping forward and lessen the labour.

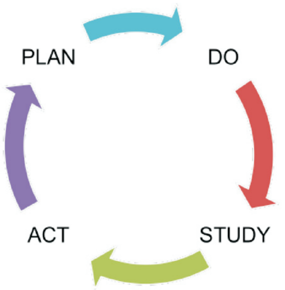

PLAN, make a clear plan for actions targeting a certain problem including assigning who is responsible, a deadline for undertaking the planned action and an expected outcome of the action.

DO, to carry out the planned action;

STUDY, to compare the expected outcome of the undertaken action with the result of the action, and;

ACT, to reflct and discuss the lessons learnt relating to the action and its outcome including taking decisions on refnements, adjustments or how to support the action to become routine.

***Figure 1:* The Plan-Do-Study-Act cycle**

Successful implementation of new practices is the achievement of agreed goals. There are four major things to consider to achieve successful implementation, namely: the new practice to be implemented, the recipients whom will adopt the new practice, the context in which the new practice is implemented and facilitation as the active ingredient that is used to integrate the three other components. The facilitator thereby holds an essential role in understanding the other three and how they interact.

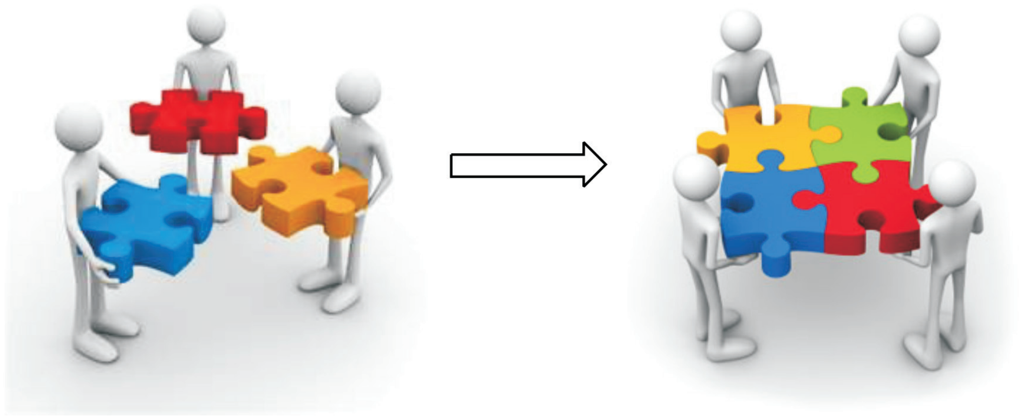

***Figure 2:* The facilitator's role is to support groups in jointly sharing experience and expertise and to work together to improve quality of care**

7. Fundamentals for QI

For the effective implementation of QI processes for perinatal care, the following four fundamental aspects should be functional;

Structure for implementation of QI process	Standards of perinatal care	Process/system in place	Mechanism for measurement of change

7.1. Structure for implementation of QI process

The intervention for Quality Improvement of perinatal care will build upon the existing structure at public hospitals. At regional and zonal level hospitals existing Maternal and Perinatal Death Review (MPDR) Committee will be responsible for overall coordination and oversight of QI process. Similarly, QI committee will coordinate the overall QI process implementation at lower level hospitals. In each hospital 2 to 4 QI facilitators will be identified for the facilitation of QI process. The QI process will be implemented by all service providers in each unit related to perinatal care in hospitals.

Structure for QI process implementation
1. QI committee at district hospital/ MPDR Committee at Regional and Zonal hospitals.
2. QI Facilitators (2-4) (*internal*)
3. Hospital Unit Staffs

7.2. Standards for Perinatal Care

Based on WHO standards for improving quality of maternal and newborn care in health facilities, the following standards (Table 1) related to perinatal care quality will be considered for improvement of perinatal care:

Table 1: Standards for improvement of perinatal care

**Standard 1: Every woman and newborn receives routine, evidence-based care and management of complications during labor, childbirth and the early postnatal period according to WHO guidelines**
Quality statement 1.1: Newborns receive routine care immediately after birth
Quality statement 1.2: Newborns receive routine postnatal care
Quality statement 1.3: Newborns who are not breathing spontaneously receive appropriate stimulation and resuscitation with a bag-and-mask within 1 minute of birth, according to WHO standards
Quality statement 1.4: Preterm and small babies receive appropriate care, according to WHO guidelines
Quality Statement 1.5: Newborns with suspected infection or risk factors for infection are promptly given antibiotic treatment, according to WHO guidelines
Quality statement 1.8: All women and newborns receive care according to standard precautions for preventing hospital-acquired infections.
Quality statement 1.9: No newborn is subjected to unnecessary or harmful practices during childbirth and the early postnatal period.
**Standard 2: The health information system enables use of data to ensure early, appropriate action to improve the care of every woman and newborn**

Quality statement 1: Every health facility has a mechanism for data collection, analysis and feedback as part of its activities for monitoring and improving performance around the time of childbirth.
**Standard 3: For every woman and newborn, competent, motivated staffs are consistently available to provide routine care and manage complications**
Quality statement 3.1: The skilled birth attendants and support staff have appropriate competence and skills to meet requirements during labor, childbirth and the early postnatal period.
**Standard 4: The health facility has an appropriate physical environment, with adequate water, sanitation and energy supplies, medicines, supplies and equipment for routine maternal and newborn care and management of complications**
Quality statement 4.1: Water, energy, sanitation, hand hygiene and waste disposal facilities are functional, reliable, safe and sufficient for the needs of staff, women and their families.
**Quality statement 4.2: Areas of labor, childbirth and postnatal care are designed, organized and maintained so that every woman and newborn can be cared for according to their needs in private, to facilitate the continuity of care**
Quality statement 4.3: Adequate stocks of medicines, supplies and equipment are available for routine care and management of complications.

7.3. Process/ System

In each of the hospitals, the Quality Improvement intervention will entail the following major process (Table 2):

Table 2. Processes of Quality Improvement intervention.

1. Assessment of service readiness, availability and quality of perinatal care.
2. On-site planning for implementation of QI for perinatal care.
3. Implementation of QI processes and plan
4. Periodic review of QI process by internal teams
5. Continuous assessment of quality of perinatal care and QI process implementation

7.4. Measurement of change

The changes observed through QI process implementation will be measured using progress boards. Also the changes will be measured through continuous assessment of quality of newborn care and QI process implementation during the third phase- ‘sustaining the change’.

The Quality Improvement interventions across the structure, standards, process and mechanism for measurement of change have been illustrated through following schematic flow diagram:

Schematic Flow for Quality Improvement of Perinatal Care

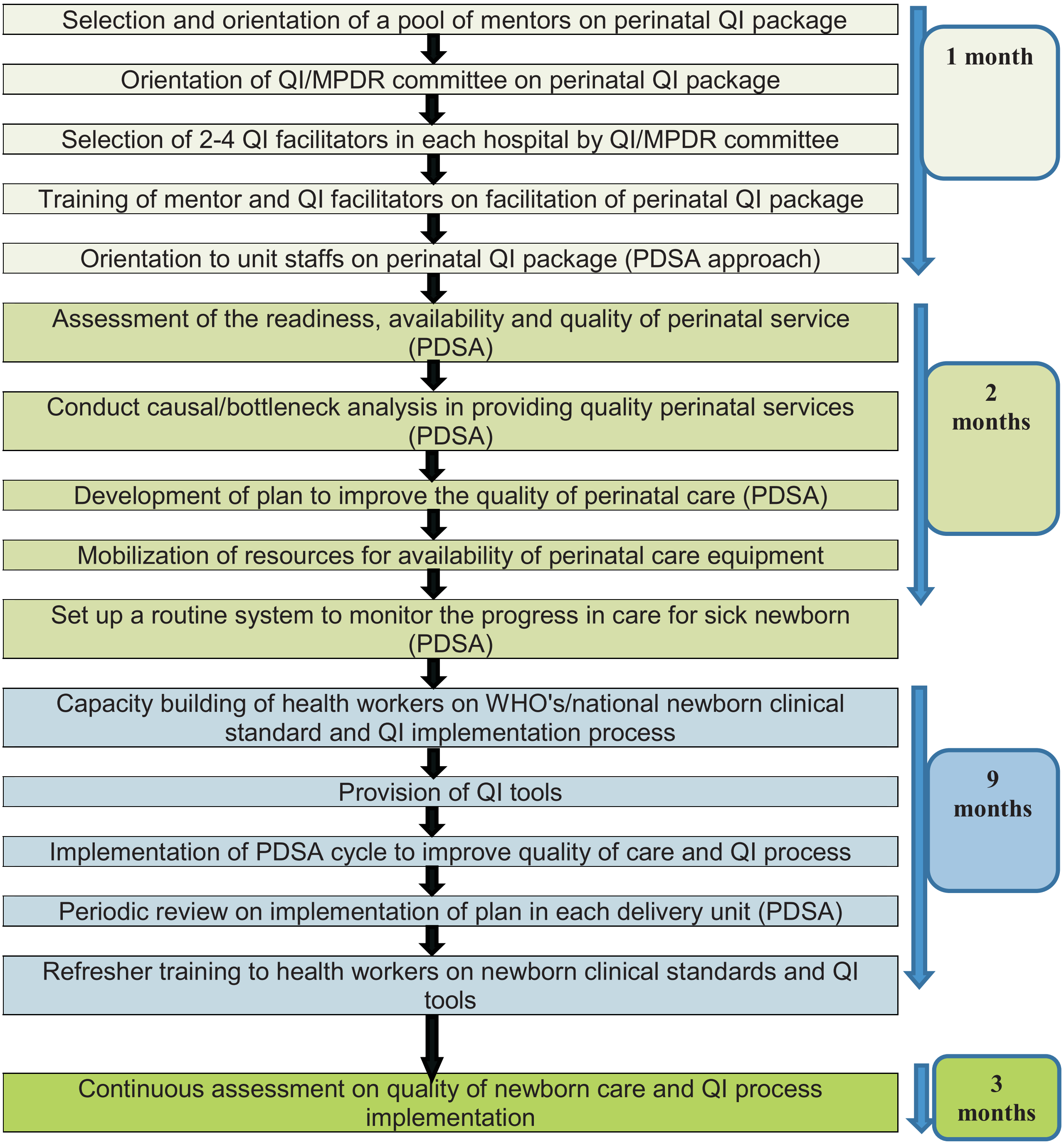

***Figure 3:* Schematic flow for Quality Improvement of Perinatal Care**

Note: *The interventions have been described in the section implementation approach*

8. Implementation Strategies

The QI interventions will utilize a combination of three different implementation strategies (1) Facilitation, (2) Process Audit and Feedback, and (3) Training, with the aim to strengthen the health care system through improved quality improvement processes and information systems, and thereby improving quality of perinatal care. Each strategy will have different components as described in Table 3. Components may overlap and will be delivered as a package. For example at unit meetings, that will happen weekly during the first three months of implementation phase and then monthly, progress boards or daily skill checks might be used to initiate discussions.

Table 3. Implementation strategies and corresponding Quality Improvement intervention

Implementation strategies	Strategy components	QI interventions/Activities
**1. Facilitation**	1.1. Plan-Do-Study-Act (PDSA) methodology	• Revitalizing of current QI processes and MPDR structures<br>• Unit meetings
	1.2. Holistic and task-oriented facilitation	• Unit meetings<br>• Training sessions
	1.3. Regular supervision and support of facilitators	• Mentoring sessions with facilitators 3 days/month<br>• Supervision of facilitators' performance by mentors
	1.4. Needs-based in-house training	• Individual training of facilitators by mentors
	1.5. Experience sharing	• Facilitator web-based interaction
**2. Training**	2.1. Training of trainers	• Training of facilitators and mentors (Training of Trainers)
	2.2. In-service training of health workers	• Initial training of health workers.<br>• Refresher trainings after six months
**3. Process Audit and Feedback**	3.1. Readiness assessment	• Survey performed during preparatory phase<br>• Dissemination of results within health system
	3.2. Peer evaluation	• Skill checks with peer evaluation<br>• Performance evaluation using checklists to be discussed with peers
	3.3. Self evaluation	• Daily individual skill checks<br>• Individual performance assessments after delivery
	3.4. Progress tracking	• Daily compilation of data displayed on progress boards

9. Implementation Approach

This guide aims to introduce and orient stakeholders to QI of perinatal care including provision of practical guidance on how it should be initiated and run. The QI of perinatal care will be implemented in following three phase (*as illustrated in figure 3*).

9.1. Preparatory phase

Following major intervention steps will be under-taken during the Preparatory phase.

Step1. Selection and orientation of mentors.

'Mentor' is the person responsible for providing all necessary technical support to hospitals for the effective implementation of QI process. A pool of mentors will be selected by Child Health Division based on defined criteria. After selection, mentors will be oriented on QI process and skill standardization before assigning the task.

Description

**Objective**	To create a pool of external mentors to provide technical support to hospitals for overall quality improvement process (assessment, capacity building of health workers, implementation of QI process, periodic review of progress)
**Responsible**	Child Health Division
**Facilitator**	IMNCI Officer
**Where**	Kathmandu
**Activity**	▪ Selection and placement of mentors based on defined criteria<br>▪ Orientation of mentors on QI implementation process focusing;<br>• QI process (mentoring)<br>• Skill standardization (clinical + facilitation/communication)
**Time**	1st week of preparatory phase
**Criteria for selection**	▪ Pediatrician/ Nurse midwives<br>▪ Actively engaged on or working on neonatal care (> 2 years).<br>▪ Well motivated to work as a mentor.<br>▪ Good interpersonal and facilitation skills.

****note:** see annex 2 (page 27) for role of Mentors*

Step 2: Orientation of QI/MPDR committee on perinatal QI package

The members of hospital QI Committee or MPDR Committee will be oriented on perinatal QI package in coordination with Child Health Division and Family Health Division at Kathmandu. The hospital QI Committee or MPDR Committee will be oriented on:

- The global, national and sub-national context of perinatal health and evidence-based perinatal care practices.
- How to improve perinatal care including the importance of multi-disciplinary involvement, the role of facilitators in change management and the use of the PDSA cycle to accomplish change.
- How QI/MPDR committee members can contribute to a supportive environment for change.

- What are the possible ways to create change and sustain it (including resource mobilization with engagement of hospital development committee).
- Criteria and process for QI facilitator selection including ToR.
- Recording and reporting QI data.

Description

**Objective**	• To orient members of QI committee at district hospitals and MPDR committee at regional/ sub-regional or zonal hospital on implementation approach of QI for perinatal care.<br>• To develop common understanding amongst the managers from different hospitals on implementation approach of perinatal care quality improvement.<br>• To orient QI/ MPDR committee members on different tools to be used for preparation and implementation of QI.<br>• To share local experience of perinatal care practices among managers from different hospitals.
**Responsible**	Mentors
**Facilitator**	IMNCI officer, CHD
**Resource Person**	CHD representative (Director, IMNCI section chief)
**Where**	Respective hospitals
**Activity**	• Provide the global, national and sub-national context of perinatal care<br>• Provide evidence on how to improve perinatal care and importance of a multi-disciplinary approach for change<br>• Develop criteria to select QI facilitators from within the hospital.<br>• Develop a plan of action to implement QI for perinatal care
**Time**	2nd week of preparatory phase
**Duration**	2 days
**Note to the facilitator**	• Pre-inform the QI /MPDR committee members and resource persons from Child Health Division about the agenda, date and venue of the orientation<br>• Prepare required logistics<br>• Prepare the presentation to the QI/MPDR committee members (based on the implementation guideline; need of QI, components of QI, approach, implementation phases, role of QI/MPDR Committee etc.)<br>• Prepare tools for selection criteria of QI facilitators and plan of action.<br>• Representative from CHD will facilitate the process.<br>• The QI/MPDR committee will identify the QI facilitator immediately after the orientation.

Step 3: Selection of QI facilitators

Depending upon the volume of delivery, 2 to 4 QI facilitators will be selected from among Pediatrician, Medical Officers and Nurses working for perinatal care in each hospital to facilitate the QI process effectively. The QI facilitators will be selected by QI/MPDR committee based on the criteria developed during orientation (step 1).

Description

**Objectives**	• To create a pool of QI facilitators for perinatal care for implementation in the hospital.
**Participants**	2 to 4 Internal Facilitators in each hospital from among Paediatrician/Medical Officer/Nursing staffs.
**Responsible**	QI/MPDR committee.
**Facilitator**	Mentor
**Where**	Respective hospital
**Activity**	• Arrange meeting for selection of QI Facilitator<br>• Select QI facilitators based on previously developed criteria through consultative process.<br>• Assign tasks to QI facilitators<br>• Communicate the roles of QI facilitators to other health workers.
**Time**	During 3rd week of preparatory phase
**Duration**	1 Day.

Note: *see annex 2 (page 27) for role of QI facilitators*

Step 4: Training of mentors and QI facilitators.

Seven days training will be provided to mentors and QI facilitators on following areas;

- Facilitator's role and the use of different strategies to engage multi-disciplinary teams in change in clinical practice.
- Training in the adoption of the PDSA cycle.
- Training of Trainers sessions on evidence-based perinatal care practices including neonatal resuscitation, kangaroo-mother care, breast-feeding, infection prevention, sick newborn care management.

Description

**Objective**	• To create a pool of competent trainers on QI of neonatal care for implementation in the hospital.<br>• To enhance the capacity of Mentors and QI Facilitators in implementing QI for neonatal care.
**Participants**	• Internal facilitators from hospital (2-4 from each hospital)<br>• Mentors (3)
**Responsible**	Child Health Division
**Resource Person**	CHD representative, representative from UNICEF/WHO
**Facilitator**	Trainer on perinatal care, facilitation techniques.
**Where**	Training Center

**Activity**	• Develop agenda, identify venue and inform participants and facilitators.<br>• Conduct training focusing on following;<br>▪ Neonatal resuscitation, Essential newborn care; KMC, Breast feeding, Infection prevention and management.<br>▪ Clinical standards of neonatal care.<br>▪ Implementation process of QI plan.<br>▪ QI tools<br>▪ Facilitation techniques.<br>▪ Tools and techniques of Health facility assessment.
**Time**	During 4th week of preparatory phase
**Duration**	7 days
**Note for facilitator**	Three to four weeks before a course is due to begin; CHD will finalize names of the participants nominated by Hospital. CHD sends out invitations to the Office of Regional Health Director with a copy to the concerned Hospital/D(P)HO, CHD and stakeholder.<br>• CHD will send the letter to concerned hospital with CC to RHD to select potential participants with Name, designation and working field.<br>• Concerned hospital will select 2-4 participants (QI Facilitators).<br>• Send request letter to Child Health Division for endorsement of the selected participants with CC to concerned RHD, RHTC and NHTC from concerned Hospital.

*(**Note:** Standard package will be developed for this training)*

Step 5: Orientation to unit staffs on perinatal QI package

One day orientation program will be organized for staffs working in different units related to neonatal care (delivery, NICU/SNCU, emergency, OPD) in hospitals. The orientation will be focused on implementation approach of newborn QI package.

Description

**Objective**	• To orient health workers at hospitals on implementation approach of QI for perinatal care.<br>• To develop common understanding amongst the health workers from different units to implement QI for perinatal care.<br>• To share local experience of neonatal care practices among managers from different hospitals
**Responsible**	QI Facilitators
**Facilitator**	Mentors
**Resource Person**	CHD representative (Director, IMNCI section chief)
**Where**	Training hall of respective hospitals
**Activity**	▪ Provide the global, national and sub-national context of perinatal care<br>▪ Provide evidence on how to improve perinatal care and importance of a multi-disciplinary approach for change
**Time**	5th week of preparatory phase
**Duration**	1 day

Step 6: Assessment of the readiness, availability and quality of perinatal care services.

An assessment of the context in which the QI for perinatal care will be implemented will be undertaken as part of the Preparatory phase. The assessment will be undertaken by external assessors (Mentor) and QI facilitators and will include assessment of essential equipment for perinatal health care practices and provision of lacking equipment.

Description

**Objective**	To assess the hospital readiness and availability on perinatal guideline/protocol, human resources, infrastructure, equipment, drugs, accessories, data management and service delivery.
**Responsible**	QI/ MPDR Committee
**Facilitator**	QI facilitator together with mentors
**Where**	Delivery room, Emergency, pediatric OPD, sick newborn care unit (NICU/SNCU), and medical record unit
**Activity**	• Review and adaption of the tools on self-assessment of the health facility's readiness and availability for perinatal care by the QI/MPDR Committee.<br>• Collection of the required information on preparation for self-assessment- human resource, data management, service delivery and logistics<br>• Conduct the self-assessment of the hospital using standard tools (annex 1)<br>▪ Observe infrastructure of the health facility as per the required standards for newborn care.<br>▪ Observe equipment and drugs in the delivery room and sick newborn care unit, emergency, OPD as per required standards.<br>▪ Review required document on human resource to assess the human resource adequacy.<br>▪ Interview with the hospital administrator and team on the human resource management.<br>▪ Review the client record file to assess information on the service delivered to newborn.<br>▪ Collect the data management process by reviewing the registers in the admission, delivery room and sick newborn care unit.<br>• Summarize the findings of the self-assessment as per the format and make a dashboard of the status of the service readiness and availability for perinatal care
**Time**	During the sixth week of the preparatory phase
**Duration**	2 days

**Note to the facilitator**	• For this activity, QI facilitator will build conducive environment by establishing relationship with hospital Director, matron, pediatrician, nursing in-charge and medical record officer/ medical recorder.<br>• Debriefing meeting with concerned personnel on purpose and objective of health facility readiness and service availability assessment to implement QI for perinatal care will be organized. In the debriefing meeting, QI Facilitator will set date for orientation on assessment tools getting consent from all.<br>• Assessment tools will be distributed to all concerned personnel prior to the orientation.<br>• While conducting data collection, Data collecting person should strictly follow the instruction given in each sections of the assessment tool.<br>• After completing the data collection, QI facilitator should analyze the data.<br>• Share the findings with the hospital director and other relevant hospital persons.

Step 6.2. Self-assessment of quality of perinatal care

**Objective**	To self-assess the quality of perinatal care in the hospital
**Responsible**	QI/ MPDR Committee
**Facilitator**	QI facilitator
**Where**	Delivery room
**Activity**	• Review and adaption of the clinical observation checklist to assess the care at the time of birth including resuscitation<br>• Conduct the self-assessment using the checklists (annex 3-QI tools)<br>• Summarize the findings of the self-assessment as per the format and make a dashboard on quality of care.
**Time**	During the 6th week of preparatory phase
**Duration**	2 Days
**Note to the facilitator**	• QI facilitator is required to build favorable environment by establishing relationship with delivery room staff.<br>• Set meeting with delivery room staff to internalize the existing record keeping practices.<br>• QI facilitator and mentors should observe birth preparation, care of baby and resuscitation of baby from service provider at delivery room and keep records by filling checklist given in Annex 3.<br>• QI facilitator is responsible to review the documentation of birth preparation, care of baby and resuscitation of baby during the preparatory phase.

Step 7: Conduct causal/ bottleneck analysis in providing quality perinatal care services

Based on the findings of assessment of readiness, availability and quality of perinatal care major issues/problems related to quality of perinatal care services will be identified in each hospital. Causal/bottleneck analysis will be performed to identify major causes/bottlenecks of each existing issue/problem. The process will be facilitated by QI facilitators with the technical support of mentors.

Description

**Objective**	To conduct a review on the findings of the self-assessment of service readiness, availability and quality of perinatal care and identify the cause of situation and options to improve the situation
**Responsible**	QI/ MPDR Committee
**Facilitator**	QI facilitator
**Where**	Hospital
**Activity**	▪ The QI facilitators will prepare a summary of the finding on the service readiness, availability and quality of perinatal care<br>▪ The QI/ MPDR committee will conduct a workshop/meeting with the health workers from the delivery unit, sick newborn care unit (NICU/SNCU), emergency, pediatric OPD to share the findings of the self-assessment.<br>▪ The QI/MPDR committee will probe with the health workers on the reason "WHY-WHY" behind the readiness, availability and quality of care.<br>▪ A detail matrix on the causal analysis will be developed by the multi-disciplinary team with the health worker (tool)
**Time**	During the Eighth week of Preparatory Phase.
**Duration**	2 days.
**Note to the facilitator**	▪ QI facilitator should analyze data working closely with medical recorder and sick newborn care unit (NICU/SNCU) head on readiness and availability of neonatal care services including resuscitation and its quality.

(Separate guide will be developed to conduct causal/bottleneck analysis)

Step 8: Development of on-site plan to improve the quality of perinatal care.

The quality improvement plan (P-D-S-A cycle) will be developed through consultative process in each hospital during this stage. The QI facilitator will facilitate the process of developing on-site plan with the support of QI / MPDR committee and external mentors. All perinatal care service providers working in delivery unit, NICU/SNCU, emergency and OPD will be involved during the process. The plan will be based on causal analysis/bottleneck analysis for gap in service delivery.

Based on this plan, the internal team of health workers in each unit will meet twice a month and work using the PDSA cycle during implementation phase. In addition to the PDSA meetings, the facilitators will also set-up a system for:

- Daily skill checks
- Checklist
- Scoreboards
- Progress meeting at grand rounds

Description

**Objective**	To develop the quality improvement plan (PDSA) for implementation of Quality Improvement based on the causal analysis for gap in service delivery
**Responsible**	Team of health workers (delivery unit, sick newborn care unit, emergency, OPD)
**Facilitator**	QI facilitator supported by Mentor
**Where**	Hospital
**Activity**	▪ Review of the detail matrix on the causal analysis of the current situation of service readiness, availability and quality of care by the QI/MPDR committee and QI Facilitator<br>▪ The QI/MPDR committee will probe with health workers including multi-disciplinary team on why to improve the service readiness, readiness and quality of perinatal care.<br>▪ The QI/MPDR committee will probe with health workers on why to improve the service readiness, availability and quality care for neonatal resuscitation<br>▪ How to improve Service readiness-equipment<br>▪ How to improve service availability-training<br>▪ How to improve quality of care for neonatal resuscitation<br>▪ The QI/MPDR committee will probe with health workers on how to measure the progress in service readiness, availability and quality of care<br>▪ The QI facilitators will probe with health workers on how to review the progress in implementation of the quality improvement plan.<br><br>▪ The team will have a following final product<br>o Goal of quality improvement cycle<br>o Objective of the quality improvement cycle<br>o Standards of quality improvement cycle<br>o Quality improvement process to implement the standards<br>o Progress monitoring of the quality improvement process implementation.<br>o Responsibilities of health workers during implementation of quality improvement cycle.
**Time**	During 9th week of preparatory phase
**Duration**	2 days
**Note for facilitator**	• Ensure the goal, objective for improving perinatal care service including resuscitation.<br>• Ensure the development of the standards for quality improvement cycle<br>• Ensure the quality improvement process to implement the standards<br>• Ensure progress monitoring of quality improvement process implementation

(Separate guide will be developed for onsite planning)

Step 9: Mobilization of resources for availability of perinatal care equipment.

Based on the gap identified through assessment, the QI facilitator will prepare list of required perinatal care equipment and share with QI/ MPDR committee. The QI/MPDR committee will in turn inform Hospital Management Committee about existing gap in perinatal care equipment. The Hospital Management Committee will mobilize internal resources or coordinate with Child Health Division/Family Health Division to ensure the availability of all newborn care equipment at hospital.

Description

**Objective**	To ensure availability of essential perinatal care equipment based on the readiness of change assessment in the delivery room and sick newborn care unit (NICU/SNCU).
**Responsible**	QI/ MPDR committee.
**Facilitator**	QI facilitator
**Where**	Delivery room, NICU/SNCU
**Activity**	▪ List the equipment required for quality improvement of newborn care services in the hospital based on readiness for change assessment.<br>▪ Hospital management will mobilize internal resource or identify the external resources for required equipment to delivery room and NICU/ SNCU
**Time**	During the 10th week of the preparatory phase
**Note to the facilitator**	• Prepare the list of required equipment to hospital<br>Coordinate with Child Health Division and hospital management for the timely supply of essential equipment

Step 10: Set up routine system to monitor the progress in care for sick newborn.

In-patient sick-newborn register will be functionalized in pediatric ward and NICU/SNCU. The service providers will be capacitated in using in-patient sick newborn register and the progress will be monitored.

**Objective**	To establish a routine information recording system for sick newborn in the NICU/SNCU unit through use of in-patient sick newborn care register and monthly compilation, use and reporting of the service statistics
**Responsible**	QI/ MPDR committee
**Facilitator**	QI facilitator, Mentor
**Where**	NICU/SNCU
**Activity**	▪ To orient to the doctors, nurses and health workers on the objective and importance of having routine information recording system in the special newborn care unit<br>▪ Orientation to the doctors, nurses and health workers in the NICU/SNCU on the recording of the information in the registration and reporting on a monthly basis<br>▪ Recording of the in-hospital sick newborn care in the register and dash board.<br>▪ Reporting of the in-hospital sick newborn on a monthly basis.
**Time**	During the 11th week of preparatory phase
**Note to facilitator**	• Provision of the in-hospital sick newborn care registers and reporting forms to the special newborn care unit staff<br>• Provide mentoring support to staff to fill up the register

9.2. Implementation phase

During this phase, the QI facilitators will be responsible for running the QI for perinatal care mentored by external mentors who will support them to develop in their roles. The QI facilitators will train their colleagues who are involved in provision of perinatal health care services; neonatal resuscitation, essential newborn care, kangaroo mother care, breast-feeding and infection prevention and management.

The major intervention steps involved in the implementation phase are:

Step 11. Capacity building of health workers on WHO's/national newborn clinical standard and QI implementation process

**Objective**	To enhance capacity of health workers on clinical standards of neonatal care, QI implementation and QI tools.
**Responsible**	QI facilitators
**Facilitator**	Mentors
**Participants**	All health workers in delivery unit, NICU/SNCU, emergency, pediatric OPD
**Where**	Hospital (on-site)
**Activity**	• Development of session plan including clinical exercise<br>• Arrangement of logistics, time and venue for training<br>• Conduction of trainer preparation workshop with the support of mentor as trainer.
**Time**	During the first week of implementation phase
**Duration**	3 days
**Note to the facilitator**	• After completion of training, internal facilitator needs to plan for the cascade training on clinical standards of neonatal care with QI Committee/MPDR Committee.<br>• Coordinate with hospital Director, Matron, Nursing In-charge for the selection of participants working in different units of the hospital<br>• The QI Facilitators will conduct cascade training in hospital as per the standard training norms.<br>• Mentors will support and supervise the training.<br>• Duration of the training will be of 3 days:<br>- Day 1 in accordance with national neonatal clinical protocol that will cover knowledge and skill update on neonatal clinical standard.<br>- Day 2 will address QIC implementation process.<br>- Day 3 will focus on QI tools<br>• During the training, trainer will fill the participants' evaluation checklist after each module.<br>• Preparation for training materials

Step 12. Provision of QI tools

**Objective**	• To ensure availability of QI tools at hospital
**Responsible**	QI Facilitator
**Facilitator**	Mentor
**Where**	Delivery room, NICU/SNCU
**Activity**	• Provision of the following equipment/tools<br>○ QI chart<br>○ HBB 2.0 Job-aid<br>○ Self-assessment checklist<br>○ Peer evaluation checklist<br>○ Table for skill check for bag-and-mask<br>○ HBB 2.0 Mannequin set for skill check<br>○ Bag-and-mask for resuscitation<br>○ Progress board<br>○ Bi-weekly review meeting note
**Time**	During the first week of implementation phase (combined with step 11)
**Duration**	1 day
**Note for facilitators**	• Collect all the equipment/materials for QI implementation<br>• Distribute the equipment/materials to all delivery room, NICU/SNCU<br>• Orient the health workers on use of the equipment/materials

Step 13. Implementation of PDSA cycle to improve quality of care and QI processes.

**Objective**	To implement the QI process for perinatal care.
**Responsible**	Health workers
**Facilitator**	QI facilitator
**Where**	Delivery room, NICU/SNCU, KMC units
**Activity**	• Health worker will conduct daily bag-and-mask skill check in the mannequin.<br>• Health worker will fill in the self-evaluation checklist after birth of baby<br>• Health workers will fill in the peer-evaluation checklist after resuscitation of each baby<br>• Health workers will prepare for resuscitation before every birth<br>• Health workers will fill up score (progress) board on a daily basis
**Time**	During the whole QI implementation phase and QI sustainability phase
**Note for facilitator**	• The QI facilitators will orient the health workers in the delivery room and sick newborn unit on the steps of QI process<br>• The health workers will implement the QI process

Step 14. Unit meetings (PDSA meetings)

**Objective**	To promote continuous learning and action for improved quality of maternal and newborn care through testing fit-for purpose solutions of jointly identified problems.
**Responsible**	Health workers in each units (delivery, NICU/SNCU, KMC)
**Facilitator**	QI facilitator
**Where**	Delivery room, NICU/SNCU, KMC
**Activity**	• The team of health workers will conduct review on the implementation of the QI process<br>• On a bi- weekly basis, the nursing in-charge will present the progress made in QI process implementation to the team of health workers<br>• On a bi-weekly basis, the team of health workers will conduct unit meeting to discuss on the progress of QI implementation and challenges in implementing the QI processes.<br>• The QI facilitator will support a group of recipients involved in perinatal care in accordance with the neonatal health and its possible solutions.<br>• The group of health workers will develop a plan for testing the solutions (Plan), carry out the test (Do), observe and learn from the consequences (Study) and determine what modifications should be made to test (Act). The<br>• The QI facilitators will brief the progress to QI/ MPDR Committee on a monthly basis.
**Frequency**	• Bi-weekly during first three months of implementation phase<br>• Twice a month during remaining six months of implementation phase.
**Time**	During the QI implementation and QI sustainability phase
**Duration**	Half day
**Note to the facilitator**	• The facilitator will set a date and time for the weekly review meetings<br>• The facilitator will take note on the proceedings of the meeting.

Step 15. Refresher training to health workers on standard of care and QI process/ tools.

**Objective**	To update health workers on national clinical standard of neonatal care and QI process/ tools.
**Responsible**	QI facilitator
**Facilitator**	Mentor, Trainers on QI for perinatal care.
**Where**	Training hall in the hospital
**Activity**	• Refresher training will be conducted in the hospital to health workers and Mid-wives working in delivery room, NICU/SNCU, emergency, pediatric OPD who are previously trained in QI for perinatal care.<br>• The Mentors/QI facilitators will be the trainers.<br>• The refresher training will be of one day on QI for neonatal care (half day clinical standard + half day on QI implementation)<br>• The trainer will fill participants evaluation checklist to access the skills acquire by the participants.<br>• To make cascade training effective and friendly, a checklist will be used during the training

**Time**	Six months after initial training
**Note for the facilitator**	• The facilitator together with team of health workers will develop the refresher training time plan, participants list and venue<br>• The facilitator will prepare the equipment and tools for the refresher training<br>• The facilitator will organize the training

Note: *This guideline does not describe details on tool used, assessments made, conduction of the training session or operation of training sites, conduction of facilitated PDSA meetings, mentoring of facilitators and transition plan for sustainability.*

9.3. Sustaining the change

This phase will transition for the sustainability of QI for perinatal care implementation in the hospital settings with full ownership and institutionalization of the program without any external support. The QI/MPDR Committee, QI facilitators and unit staffs will ensure the continuation of the positive changes occurred during the implementation phase. The step involved during the sustainability phase is;

Step 16. Continuous assessment of the service readiness, availability and quality of newborn care.

After 10 months of implementation of the program, a joint assessment on health facility readiness, availability and quality of newborn services will be carried out to measure the progress made after introduction of perinatal QI.

**Objective**	To make a review in the change in health facilities readiness, availability and quality of newborn care in the hospital
**Responsible**	QI/MPDR Committee
**Facilitator**	QI facilitator
**Where**	Hospital
**Activity**	• The QI facilitator will be responsible to conduct the health facility assessment on service readiness, availability and quality of perinatal care using the same tool used during the preparation phase.<br>• QI facilitator will have to follow all the steps of assessment as done in the baseline.<br>• The QI facilitator will work in close coordination with multi-disciplinary team for any issues and gaps for the further sustainability of the program<br>• The QI/MPDR committee, External mentor, QI facilitator will assess on change in performance of health workers on perinatal care including resuscitation, and change in quality of care focusing on birth preparation and care of baby at birth.<br>• The review in the change of performance will be carried out in the delivery room.
**Time**	During the QI sustainability phase
**Note to the facilitator**	• The Facilitator will identify a date for conducting the review of the facility in consensus with the multi-disciplinary team.<br>• Facilitator will arrange all the tools required to conduct the review<br>• The facilitator and Perinatal Stakeholder Group will discuss the progress and sustainability plan of the HBB QI

Annexes

Annex 1- Checklist for assessment of service readiness and availability of Newborn Care in hospitals

Section 1	Facility Identification Information
Section 2	Availability of Services
Section 3	Human Resources
Section 4	Infrastructure, equipment and records

Note: *Tool has been developed separately for assessment.*

Annex 2- Roles and responsibility of different organizations/ personnel

Child Health Division (CHD)
• Identify national service needs and sites/cadre to be trained (in collaboration with FHD, Hospital, D(P)HO, RHD, RHTC and NHTC)<br>• Facilitate posting/retention of needed staff for at least 3-5 years<br>• Monitor quality of services at service sites identified for training<br>• Update Technical working group about the progress.<br>• Ensure the physical facilities to provide quality services as staffs trained and graduates.<br>• Ensure availability of the equipment and instrument at the service site where service provider has been trained
**Family Health Division**
• Identify national service needs and sites/cadre to be trained (in collaboration with CHD, Hospital, D(P)HO, RHD, RHTC and NHTC).<br>• Facilitate posting/retention of needed staff for at least 3-5 years.<br>• Monitor quality of services at service sites identified for training.<br>• Update Technical working group about the progress.<br>• Ensure the physical facilities to provide quality services as staffs trained and graduates.<br>• Ensure availability of the equipment and instrument at the service site where service provider has been trained.
**Regional Health Directorate (RHD)**
• Facilitate identification and release of appropriate candidates for training based on established criteria.<br>• Assist CHD and NHTC or other concerned division/centres to identify the service and training needs in coordination with Hospital/D(P)HO<br>• Facilitate posting/retention of needed staff for at least 3-5 years.<br>• Ensure services are provided in accordance with national guidelines.
**Hospital**
• Ensure services are provided in accordance with national guidelines.<br>• Ensure staff for ongoing supervision to maintain quality of services and provide support to participants post-training (Conducting follow-up and analyses and taking action accordingly).<br>• Manage replacement of personnel to ensure continued service provision in the event staff are absent for training<br>• Identify and release appropriate candidate based on criteria.<br>• Monitor/supervise the establishment of the service after training.
**Training Sites (clinical)**
• Work closely with NHTC regarding the implementation of the training.<br>• Train participants as per the standard curriculum (Training Packages).<br>• Ensure that training equipment (AV materials, models, etc.) is maintained and is in good working order.<br>• Facilitate coordination with NHTC/RHTC and key stakeholders regarding training-related issues.<br>• Keep appropriate records and prepare necessary reports for NHTC.<br>• Conduct follow-up of the trainings and submit the report to NHTC on time.

**QI Committee/ MPDR Committee.**
• Selection of QI Facilitators from the hospital.<br>• Periodic review of the progress in QI implementation and timely feedback to Internal Facilitator and Multi-disciplinary team.<br>• Coordinate for ensuring the availability of required infrastructure, logistics (equipment, drugs), basic amenities, trained human resource for newborn care.<br>• Coordinate with Child Health Division, Family Health Division, Management Division, Logistic Management Division and other concerned stakeholders for effective implementation of QI process.<br>• Create supportive environment for the implementation of QI plan in the hospital.
**QI Facilitator**
• Assessment of hospital readiness and availability of quality newborn care.<br>• Onsite planning for implementation of QI process.<br>• Provide on-site training to health workers on clinical standards for neonatal care and QI tools.<br>• Periodic review of the progress of QI progress.<br>• Ensure effective implementation of QI plan.<br>• Update QI/ MPDR Committee on progress, issues identified and possible solutions for effective implementation of QI progress.
**Mentor**
• Orient hospital QI Committee/ MPDR Committee on Quality Improvement implementation process.<br>• Assist QI Facilitators in assessment of hospital readiness and quality newborn care including relevant (perinatal) findings from MPDR onsite planning, training of health workers.<br>• Orient QI Facilitator and health workers on various tools (assessment, planning, review)<br>• Provide technical backstopping to QI Facilitator and health workers during the implementation of QI process.<br>• Orient QI Facilitator to maintain recording and reporting QI data.<br>• Coordinate with Child Health Division, Family Health Division, hospital management committee and other relevant stakeholders for effective implementation of QI at hospitals.
**Health Workers**
• Implement QI plan with the support of QI Facilitator and QI/MPDR Committee.<br>• Participate actively in review meetings<br>• Inform QI/MPDR Committee on issues, problems encountered during the implementation of QI plan.

Annex 3- Daily Observation on Newborn Care QI Tools

Tool 1: Birth preparation (Infection Prevention)

Tool 2: Care of baby at birth (Immediate Newborn Care)

Tool 3: Resuscitation of baby (Newborn Resuscitation)

Tool 4: Identification of sick newborn (Infection management)

Tool 5: Neonatal sepsis management (Infection management)

Tool 6: Kangaroo Mother Care

Tool	Total Standard	Assessment	All completed Y/N	NA Which Number
1	1-18	Birth preparation (Infection Prevention)		
2	1-10	Care of baby at birth (Immediate Newborn Care)		
3	1-8	Resuscitation of baby (Newborn Resuscitation)		
4	1-8	Identification of sick newborn (Infection Management)		
5	1-5	Neonatal Sepsis (Infection Management)		
6		Kangaroo Mother Care		

** Note: Details guideline for use of each tool has been developed separately.*

Annex 4- Training evaluation checklist

Training............................ Participant (Trainer) ………… Training Center|……….

S. N.	Description Criteria	Observation Grading* 1...................10			Feedback
		First	Second	Third	
	Contents delivered are clearly understandable				
	Contents delivered are simple				
	Queries raised by the participants are answered effectively and to the point				
	Draws attention of the participants				
	Proper use of audio visual equipment				
	Full involvement of participants				
	Training curriculum followed as spelled out				
	Helpfulness				
	Coordination				
	Body language during training				

This can be used three times for the same participants.

Note: *1=10, 2=20, 3=30, 4=40, 5=50, 6=60, 7=70, 8=80, 9=90, 10=100*

Overall feedback

....
....
....
....
....
....

Observer:

....

Annex 5- Checklist for Training Quality Improvement

Name of the Program:	
Date /÷ Place	
Internal ☐ External ☐	Name of the Observer 1... 2... 3...
Designation and Institution of Observer	1... 2... 3...

Scoring Key: Y=Yes, N=No, NA=Not Applicable

PERFORMANCE STANDARDS	DEFINITION (VERIFICATION CRITERIA)	1	2	3	4	COMMENTS
1. Appropriate training hall	Observe during the session					
	1. Well ventilated room					
	2. Sufficient light					
	3. Spacious for facilitation					
	4. Room with no external noise					
	5. Clean toilet near by					
	6. Availability of drinking water					
	Score: All “Yes”=1 point; Any “No”=0 point					

PERFORMANCE STANDARDS	DEFINITION (VERIFICATION CRITERIA)	1	2	3	4	COMMENTS
2. Availability of basic furniture/equipment required for training session	Observe well organized training hall					
	1. LCD projector					
	2. Mannequin /equipment					
	3. Arrangement of table and chair for all					
	4. Projector screen					
	5. Flip chart easel board / White board /Black Board (any one)					
	Score: All “Yes”=1 point; Any “No”=0 point					
3. Facilitator has necessary qualification	1. All facilitators have received ToT before the conduction training / as per Training Management Guideline of NHTC					
4. Facilitators are readiness for the training	1. Sessions are conducted as per the Trainer's guide using Reference manual.					
	2. Sessions are conducted by preparing relevant notes or highlighting in the Trainer's guide.					
	3. Demonstration materials are used during the session (e.g. mannequin, meta cards, flip chart etc.)					
	Score: All “Yes”=1 point; Any “No”=0 point					
5. Facilitators are delivering contents effectively	Observe during the session:					
	1. Sessions are conducted correlating previous one with up coming.					
	2. Overview of the session going to be conducted					
	3. Session conduction focusing on key contents with effective delivery					
	Score: All “Yes”=1 point; Any “No”=0 point					

PERFORMANCE STANDARDS	DEFINITION (VERIFICATION CRITERIA)	1	2	3	4	COMMENTS
6. Facilitators are delivering contents based on the training package.	Observe during the session:					
	1. Sessions are predetermined for each facilitator					
	2. Prearrangement of reference materials for particular session					
	3. Use of prearranged reference materials for particular session					
	4. Encourage participants to use reference materials for that particular session					
	Score: All “Yes”=1 point; Any “No”=0 point					
7. Facilitators are summarizing the contents at the end of each session.	Observe during the session:					
	1. Summarization and reinforce key messages at the end of each session.					
	2. Giving opportunity to raise queries to all participants					
	Score: All “Yes”=1 point; Any “No”=0 point					
8. Facilitators are conducting session effectively and following standard method of asking questions.	Observe during the session:					
	1. Speaking in audible voice					
	2. Roaming around the room drawing participants' attention					
	3. Eye contact with participants while facilitating the session					
	4. Use of A/V and other materials (e.g. mannequin, meta cards, flip chart etc.)					
	5. Give suggestions politely and repeat right answers came from participants.					
	6. Response on incorrect or partially correct answers from participants positively correcting					
	7. Conduction of training along with other activities as per trainer's guide (e.g. role play, case study, group work and exercise)					
	Score: All “Yes”=1 point; Any “No”=0 point					
9. Banner	Observe (Banner should not be placed where visual presentation are made)					
	1. Appropriate place					
	Score: All “Yes”=1 point; Any “No”=0 point					

PERFORMANCE STANDARDS	DEFINITION (VERIFICATION CRITERIA)	1	2	3	4	COMMENTS
10. Government involvement in training	Observe correspondence letters					
	1. Correspondence are made from the government					
	2. Involvement of government officials in the training					
	3. Use of government premises for the training (where applicable)					
	Score: All “Yes”=1 point; Any “No”=0 point					
11. Government officials involvement in financials activities of the training	1. Involvement of government officials (local) for the financial management of the training					
12. Involvement of training center	1. NHTC/RHTC					
	Score: All “Yes”=1 point; Any “No”=0 point					
13. Proper recording of the training	Observe records to ensure					
	1. Use of attendance as per standard guideline/filled particiapants registration form					
	2. Use of training agends as per standard guideline or training conduction guidelines					
14. Appropriate proportion of facilitators and participants	Observe number of facilitators and participants according to the training package					
	1. Number of participants per batch according to the training package					
	2. Number of facilitators per batch according to the training package					
	Score: All “Yes”=1 point; Any “No”=0 point					

PERFORMANCE STANDARDS	DEFINITION (VERIFICATION CRITERIA)	1	2	3	4	COMMENTS 1
15. Appropriate use of training materials	Observe during the sessions					
	1. Availability of trainer's guide: trainer's manual/trainer's note, and course outline etc.					
	2. Availability of reference manual (SOP, job aids, participant's handbook, tools etc.)					
	Score: All “Yes”=1 point; Any “No”=0 point					
16. Organized facilitators reflection meeting at the end of day	Observe					
	1. Review and experiences of the sessions during the day by all facilitators					
	2. Discussion on upcoming sessions and session division					
	3. Review of participant's sit plan for upcoming day					
	Score: All “Yes”=1 point; Any “No”=0 point					

Total standards: 16

Number of observation	1	2	3	4
Number of accomplished standards				
In Percentage	%	%	%	%

Annex 6 - QI Bi-Weekly unit meeting.

Meeting No. ……

Meeting attended by:	
Date:	
Time:	
Venue	
Welcome and review of last meeting:	
Agenda 1: ………………………………..	
Discussion/ Decision	
Agenda 2: ……………………………….	
Discussion/ Decision	
Agenda 3: ………………………………	
Discussion/ Decision	
Agenda 4: ……………………………….	
Discussion/ Decision<br>.	

Annex 7- QI for perinatal care Implementation Guideline matrix

Activities	Who	How	When	Where
**Phase I : Preparatory phase**				
Step 1 - Selection and orientation of Mentors	Child Health Division, Supporting partner	Selection of Mentors based on defined criteria	During the 1st week of preparation phase	Kathmandu
Step 2 - Orientation of QI/ MPDR Committee members	Mentors	• Orientation on implementation approach of QI of perinatal care, different tools to be used for preparation, implementation and sustainability of QI.<br>• Share global, national and sub-national context of perinatal care<br>• Develop common understanding amongst the managers from different hospitals to implement QI of perinatal care.	During the 2nd weeks of the preparation phase	Kathmandu
Step 3- Selection of QI Facilitators	QI/ MPDR Committee.	• Select QI facilitators based on previously developed criteria through consultative process<br>• Assign tasks to QI facilitators<br>• Communicate the roles of QI facilitators to other health workers.	During 3rd week of preparation phase	Respective hospitals.
Step 4- Training of Mentors and QI facilitators	Participants: Mentor, QI facilitators<br><br>Trainers: External advisors (newborn care, neonatal resuscitation)	Conduct training focusing on following;<br>• Helping Babies Breathe,<br>• Essential newborn care, KMC, Breast feeding,<br>• Infection prevention and management.<br>• Clinical standards of neonatal care.<br>• Implementation process of QI plan.<br>• Facilitation techniques.<br>• Tools and techniques of Health facility assessment.	During 4th week of preparatory phase	Kathmandu

Activities	Who	How	When	Where
Step 5- Orientation to unit staffs on newborn QI package	Participants: All health workers in delivery, NICU/ SNCU, emergency, OPD units.<br><br>Facilitator: Mentor, QI Facilitator	Orientation on implementation approach of QI.	During 5th week of preparatory phase	Respective hospitals
Step 6- Assessment of the readiness, availability and quality of perinatal care services.				
Step 6.1 - Self-Assessment of health facility's readiness and availability for perinatal care	QI/MPDR committee, QI Facilitators, Mentor	Orientation on tools by QI Facilitators and Mentors to QI/ MPDR committee members<br>Data collection<br>Data analysis<br>Discussion with the team	During 6th week of preparatory phase	Delivery room, sick newborn care unit, emergency, OPD
Step 6.2 - Self-assessment of quality of perinatal care	QI Facilitators	Daily observation on:<br>• preparation of birth<br>• Care of baby at birth<br>• Resuscitation of baby<br>• Identification of sick newborn<br>• Neonatal sepsis management	During sixth week of preparatory phase	Delivery room, sick newborn care unit, emergency, OPD
Step 7- Conduct a causal analysis (bottleneck analysis) on the current gap in the service delivery	QI/ MPDR Committee, QI Facilitators	• Analysis of readiness and availability of resuscitation services<br>• Analysis of quality of Neonatal resuscitation service<br>• Presentation of the findings<br>• Discussion on why is the situation<br>• Discussion on how to improve the situation	During eighth week of preparatory phase	Respective hospitals.

Activities	Who	How	When	Where
Step 8 - Development of on-site plan to improve the quality of perinatal care	Health workers (delivery, sick newborn care, emergency and OPD units)<br>QI Facilitators	1. Discussion on the current service situation<br>2. Identify on why to improve the situation (Goal and Objective)<br>3. Identify how to improve the situation (as per QI standards)<br>QI Process:<br>• Training Equipment<br>• Skill enhancement<br>• Self –Evaluation<br>• Weekly review meeting<br>• Preparation for each birth<br>4. Identify how to measure progress in QI process implementation (Progress board)	During 9th week of preparatory phase	Respective hospitals
Step 9 - Mobilization of resources for availability of perinatal care equipment	QI/MPDR Committee, QI facilitator	• List the equipment required for quality improvement of perinatal care services in the hospital based on readiness for change assessment.<br>• Mobilize internal resource or identify the external resources for required equipment to delivery room and NICU/ SNCU	During 10th week of preparatory phase.	Respective hospitals
Step 10 - Set up routine system to monitor the progress in care for sick newborn.	QI facilitator, Mentor	• Orientation on in-patient sick newborn register in NICU/SNCU<br>• Provision of in-patient sick newborn register in NICU/ SNCU	During 11th week of preparatory phase	Sick Newborn Care Unit (NICU/SNCU)

Activities	Who	How	When	Where
**Phase II- Implementation phase**				
Step 11. Capacity building of health workers on WHO's/national newborn clinical standard and QI implementation process	Participants:<br>health workers from all units related to perinatal care<br><br>Trainers: Mentor, QI facilitators<br>External Advisor as observer and	• Update health workers on clinical standards based on national neonatal clinical protocol<br>• QI implementation<br>• QI tools/ equipment<br>(Total duration :3 days)	First week of implementation phase	Training hall of respective hospitals
Step 12- Provision of QI tools		• Provision of following equipment/ tools;<br>○ QI chart<br>○ HBB 2.0 Job-aid<br>○ Self-assessment checklist<br>○ Peer evaluation checklist<br>○ Table for skill check for bag-and-mask<br>○ HBB 2.0 Mannequin set for skill check<br>○ Bag-and-mask for resuscitation<br>○ Progress board<br>○ Bi-weekly review meeting note	During 1st week of implementation phase	Delivery unit, NICU/ SNCU
Step 13- Implementation of PDSA cycle to improve quality of care and QI processes.	Health Workers,<br>QI Facilitators	• Daily bag and mask skill check<br>• Preparation for all birth<br>• Use of self - evaluation checklist<br>• Use of peer evaluation checklist<br>• Filling up of progress board	Throughout the period of implementation phase	Delivery room, Sick newborn care unit

Activities	Who	How	When	Where
Step 14 -Periodic review of plan in each unit	Health workers from all units.<br>QI Facilitators	• Bi-weekly review and reflection meeting to review progress in implementation of QI	Throughout the period of implementation phase	Delivery room
Step 15 - Refresher training to health workers on QI for perinatal care	Participants: Health Workers<br>Trainer: Mentor, QI Facilitator.	• Update health workers on neonatal care clinical standards and QI implementation	During 6th month of implementation phase.	Training hall of respective hospitals.
**Phase III- Sustainability phase**				
Step 16-Continuous assessment of the service readiness, assessment and quality of perinatal care	QI/ MPDR Committee, QI Facilitator, Mentor.	• Orientation on tools by QI Facilitators to members of QI/ MPDR Committee.<br>• Data collection: (Observe ask & review)<br>• Data analysis<br>• 4. Discussion on the gaps and plans for improvement	During phase III - Sustaining the change	Respective hospitals
