# Supplementary material for: Effect of a scaled-up quality improvement intervention on health workers’ competence on neonatal resuscitation in simulated settings in public hospitals: A pre-post study in Nepal
Source: PLoS One. 2021 Apr 29;16(4):e0250762. doi: 10.1371/journal.pone.0250762 (PMC8084235; doi:10.1371/journal.pone.0250762)
Supplement: S2 Text — (PDF) [file pone.0250762.s002.pdf]

Nepal Perinatal Quality Improvement Project (NePeriQIP)

# NePeriQIP Training Registration and Course Evaluation Form

| | |
|---|---|
| Participants No. | |
| **Location of Training** | |
| Date | |

UPPSALA UNIVERSITET

unicef

# Training Registration

| S.N | Question | Response<br>(put circle in close ended and response to open ended questions) | |
|---|---|---|---|
| 1. | Name | | |
| 2. | Family Name | | |
| 3. | Age( completed years) | | |
| 4. | Sex | Male……………………………………………….1<br>Female………………………………………...…...2 | |
| 5. | Contact phone number | | |
| 6. | Work Phone | | |
| 7. | E-mail | | |
| 8. | Hospital/clinic or Institution | Maternity Hospital………..……………………..……1<br>Regional Hospital………..….................................2<br>Zonal Hospital…………………………………….…3<br>District Hospital…………………………………...……4<br>Teaching Hospital…………………………………....5<br>Nursing College……………………………………… 6<br>Others (specify)……………………………………. | |
| 9. | Name of the institution | | |
| 10. | Address | | |
| 11. | Mobile Phone | | |
| 12. | Academic Qualification | **Student**<br>ANM……………………….………………………...……1<br>SN…………………………………………………….... 2<br>BN……………………………………………………....3<br>B.Sc. Nursing…………………..….…….…..…………….4<br>MN………………………………………..……………..…….5 | |
| | | **Nurse**<br>ANM……………….……………………………………6<br>SN…………………………………..……………………7<br>BN……………………………………..………………….. 8<br>BSc…………………………..………………………….9<br>MN……………………………..……………..…………..10 | |
| | | **Medical**<br>Student……………………………..……………...…..11<br>Intern/Resident……………………………………….. 12<br>Medical Doctor………………………………………... 13<br>Gyn/Obs……………………………………………..14<br>Pediatric……………………………..………………....15<br>Other (Specify)……………………..………………… | |

| S.N | Question | Response<br>(put circle in close ended and response to open ended questions) | |
|---|---|---|---|
| 13. | Current Post/Responsibility | Student………………………………….……………..1<br>Student Coordinator…………..…………………...2<br>Staff Nurse…………………………………….………...3<br>Sister in Charge……………………………………….4<br>Nursing Supervisor…………….………………......5<br>Medical Officer…………………..……………… 6<br>Sr. Medical Officer……………….….…………...7<br>Specialist…………………………………………………....8<br>Other (Specify)………………………………………..… .. | |
| 14. | How many years have you worked in your profession? (completed years) | ___________Years | |
| 15. | Have you ever received training in newborn resuscitation? | Yes………...………………………………………… 1<br>No…………….……………………………………………...2 | |
| 16. | If Yes, which training have you received in last two years? | ________________________________ | |
| 17. | In an average month, how many births do you attend/perform? | None………………………..……………………………..1<br>1-5…………………………..……………….……………...2<br>6-15……………………………...……………………………. 3<br>16-25………………………………………….………………...4<br>26-50…………………………...……..………………..……...5<br>More than 50…………...……………………….……………....6 | |
| 18. | Do you resuscitate newborn babies? | Yes………...………………………………………….1<br>No……………….……………………………………...….2 | |
| 19. | In the past, have you used a bag and mask to resuscitate a baby? | Yes………...……………………………………..…. 1<br>No………………….………………………………………….2 | |
| 20. | How many babies have you resuscitated with a bag and mask in the past year? | None…………………….………………..……………..1<br>1-2……………………….…………………………………….2<br>3-5……………………..…………………….…..………..….3<br>6-12……………………..…………….………..……………..4<br>More than 12….………………………..………………….5 | |
| 21. | What neonatal resuscitation equipment does your primary facility currently have? (checkall that you have in your institutions) | Resuscitator (Bag and mask)……….………………..1<br>Bulb syringe ………………………………………..………..2<br>Stethoscope…………………………………….…………...3<br>None of the above…………………….………………...99 | |
| 22. | Would you like to be member of Helping Babies Breathe Nepal Circlein Social Network (Facebook closed group) | Yes……………………..………..……………………………1<br>No…………………….…………………………...……… .2 | |
| 23. | If yes, your social network ID | | |
| 24. | Registration Fee | NRs___________<br><br>NA | |
| 25. | Signature | ________________________ | |

# HELPING BABIES BREATHE

## PRE-TEST

To record your answers, completely fill in the circle that contains the appropriate letter, as shown in the example.

Example: ⓐ ⓑ ⓒ ●

1. ⓐ ⓑ ⓒ ⓓ
2. ⓐ ⓑ ⓒ ⓓ
3. ⓐ ⓑ ⓒ ⓓ
4. ⓐ ⓑ ⓒ ⓓ
5. ⓐ ⓑ ⓒ ⓓ
6. ⓐ ⓑ ⓒ ⓓ
7. ⓐ ⓑ ⓒ ⓓ
8. ⓐ ⓑ ⓒ ⓓ
9. ⓐ ⓑ ⓒ ⓓ
10. ⓐ ⓑ ⓒ ⓓ
11. ⓐ ⓑ ⓒ ⓓ
12. ⓐ ⓑ ⓒ ⓓ
13. ⓐ ⓑ ⓒ ⓓ
14. ⓐ ⓑ ⓒ ⓓ
15. ⓐ ⓑ ⓒ ⓓ
16. ⓐ ⓑ ⓒ ⓓ
17. ⓐ ⓑ ⓒ ⓓ

## A. To evaluate the skills on preparation at birth

What are the preparations that needs to be done before birth of each baby

| Preparation for Birth | Yes | No |
|---|---|---|
| 1. Identification of helper and review of emergency plan | ▪ | ▪ |
| 2. Wash hand | ▪ | ▪ |
| 3. Prepare an area for ventilation | ▪ | ▪ |
| 4. Assemble all supplies and equipment | ▪ | ▪ |
| *(Gloves, clothes, head covering, scissors, clamps, suction device, Bag-Mask, stethoscope, timer)* | | |
| 5. Check the bag and mask for ventilation | ▪ | ▪ |

**Score on first attempt ________ of 5**

**All steps done correctly __________________ (facilitator initials)**

## B. To evaluate the skill in Helping Babies Breathe

| | Yes | No |
|---|---|---|
| 1. Check equipment and select correct mask<br>Test the function of Bag and Mask<br>Make sure mask fits the baby's face | ■ | ■ |
| 2. Apply the mask to make a firm seal<br>Extend the head, place mask on the chin, then over the mouth and nose<br>A firm seal permits chest movement when the bag is squeezed | ■ | ■ |
| 3. Ventilate at 40 breathes per minute (30-50) | ■ | ■ |
| 4. Look for chest movement<br>Check that for every ventilation breath produces chest movement | ■ | ■ |
| 5. Improves Ventilation if the chest does not move | | |
| a. Head-reapply mask and reposition head | ■ | ■ |
| b. Mouth-clear secretions and open the mouth | ■ | ■ |
| c. Bag-squeezes harder | ■ | ■ |

**Score on first attempt ________ of 7**

**All steps done correctly __________________ (facilitator initials)**

## C. To evaluate the skills in Helping Babies Breathe (OSCE 1)

**Instructions to the facilitator:**

Read aloud to the learner the following instructions and the case. Provide prompts where shown in *italics*. As you observe the learner, tick ☑ the boxes "Done" or "Not Done" for each activity. Indicate the baby's response to the learner's actions using the neonatal simulator or words. Note the time between birth and beginning ventilation. Comment on the learner's performance only at the end of the case.

"I am going to read a role play case. Please listen carefully, and then show me how you would care for this baby. I will indicate the baby's response with the simulator (OR in words). I will provide no other feedback until the end of the case." "On 2069/12/31, 7:00 pm you are called to assist at the birth of a 34 week (7 ½ months) gestation baby. You arrive at 7:10 pm two minutes prior to birth. Introduce yourself and show what you will do."

| | Done | Not Done |
|---|---|---|
| **Prepares for a birth**……………………………………………………………………… | ☐ | ☐ |
| Identifies a helper, prepares the area for delivery, cleans hands | | |
| Prepares an area for ventilation and checks equipment | | |
| *Prompt: After 2 minutes give baby to learner and say, "The amniotic fluid is clear. Show how you will care for the baby."* | | |
| Dries thoroughly and removes wet cl……………………………………………….. | ☐ | ☐ |
| **Evaluates crying.** *Prompt: Show or say the baby is not crying.* | | |
| Recognizes baby is not crying………………………………………………………… | ☐ | ☐ |
| **Clears airway and stimulates breathing** | | |
| Keeps warm, positions head, clears air….…………………………………………… | ☐ | ☐ |
| Stimulates breathing by rubbing the back ..…………………………………………. | ☐ | ☐ |
| **Evaluates breathing** | | |
| RECOGNIZES BABY IS NOT BREATHING………..…………………………………… | * ☐ | ☐ |

**Ventilates with bag and mask**

Cut cord and moves to area for ventilation OR ventilates by mother ………… ☐ ☐
Starts ventilation within The Golden Minute[SM] (at___ seconds) …………..…….. ☐ ☐
VENTILATES AT 40 BREATHS/MINUTE (30-50 acceptable) ……………… * ☐ ☐
LOOKS FOR CHEST MOVEMENT ………………………………………………… * ☐ ☐

**Evaluates breathing** *Prompt: Show or say the baby is not breathing*

Recognizes baby is not breathing………………………………………………….. ☐ ☐
Calls for help………………………………………………………………………..….. ☐ ☐
Continues ventilation……………………………………………………………………. ☐ ☐

*Prompt: Say, "Please show what to do if the chest is not moving with ventilation."*

After one or more steps to improve ventilation, say, "The chest is moving now."

**IMPROVES VENTILATION**…………………………………………………... * ☐ ☐
Head –repositions head, reapplies mask
Mouth – clears secretions, opens mouth slightly
Bag – squeezes bag harder

***Evaluates breathing and heart rate***. *Prompt: Show or say the baby is not breathing; heart rate is normal.*

Recognizes baby is not breathing but heart rate is normal ………………………. ☐ ☐
Continues Ventilation ………………………………………………………….……….. ☐ ☐

*Prompt: After 3 minutes show or say, "The heart rate is 120 per minute and the baby is breathing."*

Recognizes baby is breathing and heart rate is normal…………………………… ☐ ☐
Stops ventilation; monitors baby and communicates with mother.….. ……….. ☐ ☐

.

Scoring: Successful completion requires a total score of 14 correct of 18 and "Done" must be ticked for **RECOGNIZES BABY IS NOT BREATHING, VENTILATES AT 40 BREATHS PER MINUTE, LOOKS FOR CHEST MOVEMENT**, and **IMPROVES VENTILATION.**

Number Done Correctly____ Facilitator initials____

## D. To evaluate the skills of record keeping on immediate care of baby

Now use OSCE 1 to do the recording of the baby's clinical outcome at the time of birth

### Baby Examination sheet

| **Management of Newborn at birth** | **Yes** | **No** |
|---|---|---|
| Meconium in the baby's mouth | ▪ | ▪ |
| Drying thoroughly and removing wet clothes | ▪ | ▪ |
| **Assess:** Baby is not crying | ▪ | ▪ |
| Keep the baby warm | ▪ | ▪ |
| Clearing the airway/Stimulation of breathing | ▪ | ▪ |
| **Assess:** Baby is not breathing | ▪ | ▪ |
| Clamp and cutting of umbilical cord | ▪ | ▪ |
| Baby moved to ventilation area | ▪ | ▪ |
| Ventilate by 1 minute | ▪ | ▪ |
| Ventilation for 1 minute | ▪ | ▪ |
| Ventilation at 40 breathe per minute | ▪ | ▪ |
| **Assess:** Effective chest movement with ventilation | ▪ | ▪ |
| **Assess:** Not breathing well | ▪ | ▪ |
| Call for help | ▪ | ▪ |
| **Assess:** Heart Rate less than 100/minute | ▪ | ▪ |
| Continue ventilation | ▪ | ▪ |
| Improve ventilation | ▪ | ▪ |
| **Assess:** HR normal and baby not breathing | ▪ | ▪ |
| **Assess:** Referral if HR is slow and baby not breathing | ▪ | ▪ |
| Continue ventilation | ▪ | ▪ |
| **Assess:** Breathing well and Heart rate normal | ▪ | ▪ |
| Ventilation stops, monitoring of baby | ▪ | ▪ |
| Any malformation | ▪ | ▪ |

**Preparation for Birth**

Identification of helper and review of emergency plan ▪

Wash hand ▪

Prepare an area for ventilation ▪

Assemble all supplies and equipment ▪
*Gloves, clothes, head covering, scissors, clamps, suction device, Bag-Mask, stethoscope, timer*

Check the bag and mask for ventilation ▪

Date of birth ___/___/______

Time of birth ______________

Place of birth AR/LR/MNSC/ANC/OT

Sex of baby F/M

Gestational age in wks

Birth Weight in gm

**Clinical performance**

Apgar score at 1 minute …../10

Apgar score at 5 minute …../10

Time of initiation of BMV …………..

Liquor …………..

Rectum patent Y/N

Meconium pass Y/N

## E. Peer Evaluation Checklist

| | | |
|---|---|---|
| Date | Place | Time of Birth |
| Name of peer | | |

| **Management of Newborn at birth** | **Yes** | **No** |
|---|---|---|
| Meconium in the baby's mouth | ■ | ■ |
| Drying thoroughly and removing wet clothes | ■ | ■ |
| **Assess:** Baby is not crying | ■ | ■ |
| Keep the baby warm | ■ | ■ |
| Clearing the airway/Stimulation of breathing | ■ | ■ |
| **Assess:** Baby is not breathing | ■ | ■ |
| Clamp and cutting of umbilical cord | ■ | ■ |
| Baby moved to ventilation area | ■ | ■ |
| Ventilate by 1 minute | ■ | ■ |
| Ventilation for 1 minute | ■ | ■ |
| Ventilation at 40 breath per minute | ■ | ■ |
| **Assess:** Effective chest movement with ventilation | ■ | ■ |
| **Assess:** Not Breathing well | ■ | ■ |
| Call for help | ■ | ■ |
| **Assess:** Heart Rate less than 100/minute | ■ | ■ |
| Continue ventilation | ■ | ■ |
| Improve ventilation | ■ | ■ |
| **Assess:** HR normal and baby not breathing | ■ | ■ |
| **Assess:** Referral if HR is slow and baby not breathing | ■ | ■ |
| Continue ventilation | ■ | ■ |
| **Assess:** Breathing well and Heart rate normal | ■ | ■ |
| Ventilation stops, monitoring of baby | ■ | ■ |
| Any malformation | ■ | ■ |

## POST-TEST

To record your answers, completely fill in the circle that contains the appropriate letter, as shown in the example.

Example: ⓐ ⓑ ⓒ ●

1. ⓐ ⓑ ⓒ ⓓ
2. ⓐ ⓑ ⓒ ⓓ
3. ⓐ ⓑ ⓒ ⓓ
4. ⓐ ⓑ ⓒ ⓓ
5. ⓐ ⓑ ⓒ ⓓ
6. ⓐ ⓑ ⓒ ⓓ
7. ⓐ ⓑ ⓒ ⓓ
8. ⓐ ⓑ ⓒ ⓓ
9. ⓐ ⓑ ⓒ ⓓ
10. ⓐ ⓑ ⓒ ⓓ
11. ⓐ ⓑ ⓒ ⓓ
12. ⓐ ⓑ ⓒ ⓓ
13. ⓐ ⓑ ⓒ ⓓ
14. ⓐ ⓑ ⓒ ⓓ
15. ⓐ ⓑ ⓒ ⓓ
16. ⓐ ⓑ ⓒ ⓓ
17. ⓐ ⓑ ⓒ ⓓ

# PRE-TEST OF Kangaroo Mother Care, Breastfeeding and Infection Prevention & Management

To record your answers, completely fill in the circle that contains the appropriate letter, as shown in the example.

Example: ⓐ ⓑ ⓒ ●

1. ⓐ ⓑ ⓒ ⓓ
2. ⓐ ⓑ ⓒ ⓓ
3. ⓐ ⓑ ⓒ ⓓ
4. ⓐ ⓑ ⓒ ⓓ
5. ⓐ ⓑ ⓒ ⓓ
6. ⓐ ⓑ ⓒ ⓓ
7. ⓐ ⓑ ⓒ ⓓ
8. ⓐ ⓑ ⓒ ⓓ
9. ⓐ ⓑ ⓒ ⓓ
10. ⓐ ⓑ ⓒ ⓓ
11. ⓐ ⓑ ⓒ ⓓ
12. ⓐ ⓑ ⓒ ⓓ
13. ⓐ ⓑ ⓒ ⓓ
14. ⓐ ⓑ ⓒ ⓓ
15. ⓐ ⓑ ⓒ ⓓ

## Skill Checklist for KMC Procedure

**Scoring keys: Y=Yes, N=No, NA= Not applicable** **Score: All Yes =1, Any No =0**

| Steps | Y/N | NA |
|---|---|---|
| 1. Mother or care-giver counselled on benefits of KMC | | |
| 2. privacy for the client ensured | | |
| 3. Hand washing done before handling the newborn | | |
| 4. Baby dressed in cap, socks, nappy and front open clothes/shirt. | | |
| 5. Baby placed in between mother's breasts in upright position | | |
| 6. Baby's head turned to one side in slightly extended position | | |
| 7. Hips are flexed and abducted and arms are also flexed (frog position) | | |
| 8. Baby's abdomen is at the level of the mother's epigastrium | | |
| 9. Baby is supported underneath gluts by a sling/binder | | |
| 10. Position of head and neck (alternate neck position), airway, breathing, colour and temperature monitored | | |
| 11. Encourage mother/caregiver to sit in a comfortable position and counsel on the need to provide KMC for 24 hours if possible | | |

## Skill Checklist for Breastfeeding Assessment

**Scoring keys: Y=Yes, N=No, NA= Not applicable** **Score: All Yes =1, Any No =0**

| No. | Steps | Rating | |
|---|---|---|---|
| | | Y/N | NA |
| 1. | Greet the mother | | |
| 2. | Ask and listen to the mother | | |
| 3. | Assess whether mother is relaxed | | |
| 4. | Assess for the position of the baby | | |
| 4.1 | Baby's whole body should be well supported | | |
| 4.2 | The baby's head and body should be in a straight line. | | |
| 4.3 | Baby's body turned towards the mother with baby's abdomen touching mother's abdomen | | |
| 4.4 | Baby's nose at the level of nipple | | |
| 5 | Assess for the attachment | | |
| 5.1 | Mouth wide open | | |
| 5.2 | Chin touching the breast and nose close to breast | | |
| 5.3 | Lower lips turned outwards | | |
| 5.4 | More areola above baby's mouth than below | | |
| 6. | Assess for effective suckling - Slow deep suck with pause | | |
| 7. | Give mother practical help if needed | | |
| 8. | Thank mother for her cooperation | | |

## Skill Checklist for Expression of Breast Milk

**Scoring keys: Y=Yes, N=No, NA= Not applicable** **Score: All Yes =1, Any No =0**

| No. | Steps | Rating | |
|---|---|---|---|
| | | Y/N | NA |
| 1. | Wash hands with soap and water before expression. | | |
| 2. | Ask mother to sit comfortably | | |
| 3. | Ask mother to hold the clean container under the nipple | | |
| 4. | Helps mother to place thumb above and first finger below and behind the nipple approximately 4cm from the base of the nipple. | | |
| 5. | Ask mother to support the breast with other three fingers | | |
| 6. | Ask mother to press the breast gently slightly inwards towards the chest wall | | |
| 7. | Ask mother to press the breast between the fore-finger and thumb. Press and release, press and release. | | |
| 8. | Ask mother to avoid rubbing or sliding fingers along the skin | | |
| 9. | Ask mother to rotate the position of the thumb/finger around the breast with each compression | | |
| 10. | Ask mother to express breast milk until milk drips, then express the other breast | | |
| 11. | Ask mother to alternate between the breasts 5-6 times (20-30 minutes) | | |
| 12. | Ask mother to consider massage of breasts and use of warm compresses prior to or during expression to improve milk flow | | |

# POST-TEST OF Kangaroo Mother Care, Breastfeeding and Infection Prevention & Management

To record your answers, completely fill in the circle that contains the appropriate letter, as shown in the example.

Example: ⓐ ⓑ ⓒ ●

1. ⓐ ⓑ ⓒ ⓓ
2. ⓐ ⓑ ⓒ ⓓ
3. ⓐ ⓑ ⓒ ⓓ
4. ⓐ ⓑ ⓒ ⓓ
5. ⓐ ⓑ ⓒ ⓓ
6. ⓐ ⓑ ⓒ ⓓ
7. ⓐ ⓑ ⓒ ⓓ
8. ⓐ ⓑ ⓒ ⓓ
9. ⓐ ⓑ ⓒ ⓓ
10. ⓐ ⓑ ⓒ ⓓ
11. ⓐ ⓑ ⓒ ⓓ
12. ⓐ ⓑ ⓒ ⓓ
13. ⓐ ⓑ ⓒ ⓓ
14. ⓐ ⓑ ⓒ ⓓ
15. ⓐ ⓑ ⓒ ⓓ

## Training Program Evaluation in terms of Participant's Satisfaction

Course evaluation form for facilitator and learner

| | | Strongly disagree | Disagree | Indeterminate | Agree | Strongly agree |
|---|---|---|---|---|---|---|
| **1.** | Organization of train the trainer course | | | | | |
| A | The course was well organized (adequate time for practice) | 1 | 2 | 3 | 4 | 5 |
| B | I had enough time to learn how to lead the course | 1 | 2 | 3 | 4 | 5 |
| C | We completed the course in the time we had | 1 | 2 | 3 | 4 | 5 |
| **2.** | Teacher | | | | | |
| A | The teacher had time for my questions | 1 | 2 | 3 | 4 | 5 |
| B | The teacher listened to my questions | 1 | 2 | 3 | 4 | 5 |
| C | The teacher gave me answers | 1 | 2 | 3 | 4 | 5 |
| **3.** | Teacher efficacy | | | | | |
| A | I can explain the Golden Minute | 1 | 2 | 3 | 4 | 5 |
| B | I can explain the action plan | 1 | 2 | 3 | 4 | 5 |
| C | I have enough information to lead the course | 1 | 2 | 3 | 4 | 5 |
| **4.** | Course materials | | | | | |
| | The following course materials will help my learners: | | | | | |
| A | Learner workbook | 1 | 2 | 3 | 4 | 5 |
| B | Mannequin, bag, and mask | 1 | 2 | 3 | 4 | 5 |
| C | Flip chart | 1 | 2 | 3 | 4 | 5 |
| D | Action plan | 1 | 2 | 3 | 4 | 5 |
| E | The pictures presented in the workbook and flip charts are appropriate | 1 | 2 | 3 | 4 | 5 |
| F | The pictures in the workbook and flip charts will help me explain the action plan | 1 | 2 | 3 | 4 | 5 |
| G | Group discussions were helpful | 1 | 2 | 3 | 4 | 5 |
| H | Self-check lists were helpful | 1 | 2 | 3 | 4 | 5 |
| **5.** | Overall | | | | | |
| A | I can help a baby breathe | 1 | 2 | 3 | 4 | 5 |
| B | I want to help people learn how to help babies breathe | 1 | 2 | 3 | 4 | 5 |

## Your perception on Facilitator and ease of teaching

A semi-structured interview guide

| | |
|---|---|
| 1. | Organization of train the trainer course |
| 2. | Teacher |
| 3. | Teacher efficacy |
| 4. | Course materials |

## Knowledge Check on Helping Babies Breathe

Select the best answer to each question or statement. Mark your choice on the answer sheet provided.

1. In the first minute after birth, you should
   a. Bathe the baby
   b. Help the baby breathe
   c. Feed the baby
   d. Not touch the baby

2. To prepare for a birth
   a. You identify a helper and review the emergency plan
   b. You ask everyone but the mother to leave the area
   c. You prepare equipment only when you need it
   d. You do not need a helper

3. To prepare the area for delivery
   a. Open all the doors and windows to get fresh air
   b. A clean space for the baby will not be required
   c. Make sure the area is clean, warm, and well-lighted
   d. Keep the room temperature cold

4. Which baby can receive routine care after birth?
   a. A baby who is not breathing
   b. A baby who is gasping
   c. A baby who is crying and/or breathing well
   d. A baby who is limp

5. Routine care for a healthy baby at birth includes
   a. Drying, removing the wet cloth, and bathing the baby
   b. Drying, removing the wet cloth, and positioning the baby skin-to-skin
   c. Bathing and putting clean clothes on the baby
   d. Drying and wrapping the baby in the wet cloth

6. When should the umbilical cord be clamped or tied and cut during routine care?
   a. After the placenta is delivered
   b. Around 1-3 minutes after birth
   c. Immediately after the baby is born
   d. Before a baby has cried

7. A baby is quiet, limp and not breathing at birth. What should you do?
   a. Dry the baby thoroughly
   b. Shake the baby
   c. Throw cold water on the face
   d. Hold the baby upside down

8. A baby is born through meconium-stained amniotic fluid. Which statement is TRUE?
   a. Stimulate the baby and then clear the airway
   b. Meconium cannot be inhaled into the lungs
   c. Clear the airway before drying the baby
   d. All babies born through meconium-stained amniotic fluid can receive routine care

9. What should you do in The Golden Minute[SM]?
   a. Bathe the baby
   b. Deliver the placenta
   c. Evaluate the heart rate
   d. Help a baby breathe if necessary

10. A newborn baby is quiet, limp and not crying. The baby does not respond to steps to stimulate breathing. What should you do next?
    a. Slap the baby's back
    b. Hold the baby upside down
    c. Squeeze the baby's ribs
    d. Begin ventilation

11. Which of the following statements about ventilation with bag and mask is TRUE?
    a. The mask should cover the eyes
    b. Air should escape between the mask and face
    c. Squeeze the bag to produce gentle movement of the chest
    d. Squeeze the bag to give 80 to 100 breaths per minute

12. Which of the following signs MUST be monitored in a baby during the first few hours after birth?
    a. Length
    b. Breathing
    c. Smile
    d. Urine output

13. A baby's chest is not moving with bag and mask ventilation. What should you do?
    a. Stop ventilation
    b. Reapply the mask to get a better seal
    c. Slap the baby's back
    d. Give medicine to the baby

14. You can stop ventilation if
    a. Baby is blue and limp
    b. Baby's heart rate is 80 per minute
    c. Baby's heart rate is 120 per minute and the chest is not moving
    d. Baby's heart rate is 120 per minute and the baby is breathing or crying

15. What should you do to keep the baby warm?
    a. Open all the windows to allow warm air to circulate
    b. Give the baby a bath after birth
    b. Give the baby a bath after birth
    c. Place hot water bottles next to the baby's skin
    d. Place the baby skin-to-skin with mother

16. What should you do to keep the baby clean?
    a. Wash your hands before touching the baby and help mother wash her hands before breastfeeding
    b. Reuse the suction device before cleaning
    c. Keep the umbilical cord tightly covered
    d. Do not touch the baby

17. A newborn baby's heart rate should be:
    a. Faster than your heart rate
    b. Slower than your heart rate

# Pre-Test and Post- test questions for KMC, Breastfeeding and IP

1. Which hormone is responsible for milk secretion?
   a) Prolactin
   b) Oxytocin
   c) Glucocorticoids
   d) Thyroid hormone

2. Which hormone is responsible for milk ejection?
   a) Testosterone
   b) Oxytocin
   c) Parathyroid hormone
   d) Prolactin

3. What are the signs for Good attachment?
   a) Mouth wide open
   b) Chin touching the breast and nose close to breast
   c) Lower lips turned outwards
   d) All of the above

4. What are the signs for proper positioning?
   a) Baby's whole body should be well supported
   b) The baby's head and body should be in a straight line.
   c) Baby's body turned towards the mother with baby's abdomen touching mother's abdomen
   d) All of the above

5. Benefit of Kangaroo Mother Care is all except
   a) Increase milk production
   b) Increase baby's weight
   c) Increase chance of apnoea
   d) Reduce chances of hypothermia

6. When to discontinue KMC?
   a) Any time
   b) After 7 days
   c) Baby starts wriggling and shows uncomfortable
   d) all of the above

7. What are the different types of breast milk?
   a) Mature milk
   b) Hind milk
   c) Fore milk
   d) All of the above

8. What is the theme of World Breast Feeding Week, 2017?
   a) Breastfeeding and work, let's make it work
   b) Breastfeeding everywhere
   c) Sustaining breastfeeding together
   d) Support Breastfeeding

9. What is the single most important means of prevention of hospital acquired infection?
   a) Fumigation of room
   b) Hand washing
   c) Prophylactic antibiotics
   d) Wearing sterile gloves

10. How frequently is Cheatle Forceps autoclaved?
   a) Daily
   b) Once a week
   c) Once a month
   d) Never

11. How will you clean Stethoscope?
   a) Spirit swab
   b) Soap and water
   c) Autoclave
   d) Only water

12. This sign is for 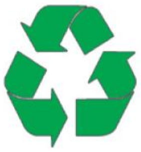

   a) Non-risk waste recyclable
   b) Traffic sign
   c) Danger sign
   d) None

13. High Level Disinfection (HLD) means;
   a) Elimination of microorganisms including bacterial endospores
   b) Elimination of microorganisms except bacterial endospores
   c) Removing sufficient number of microorganisms from skin and mucous membrane and instruments
   d) The process that makes inanimate objects safer to be handled by staffs

14. For decontamination, the instruments need to be kept in ______ solution for 10 minutes?
   a) 0.05% Chlorine
   b) 0.5% Chlorine
   c) 5% Chlorine
   d) 10% Chlorine
