# Supplementary material for: Effect of a scaled-up quality improvement intervention on health workers’ competence on neonatal resuscitation in simulated settings in public hospitals: A pre-post study in Nepal
Source: PLoS One. 2021 Apr 29;16(4):e0250762. doi: 10.1371/journal.pone.0250762 (PMC8084235; doi:10.1371/journal.pone.0250762)
Supplement: Attachment — Submitted filename: Response to reviewers.doc [file pone.0250762.s004.doc]

Anne Lee Solevåg,	25 January 2021
Academic Editor
PLOS ONE

**Re: Comments addressed for the manuscript "Effect of a scaled-up quality improvement intervention on health workers' competence on neonatal resuscitation in simulated settings in public hospitals: a pre-post study in Nepal"**

Dear Anne Lee,

Thank you for providing an opportunity to revise our manuscript with your valuable comments. We have attempted to address all comments in our revised manuscript. Please find the responses **(in bold)** to each comment below;

**Journal Requirements**

1. Please ensure that your manuscript meets PLOS ONE's style requirements, including those for file naming. The PLOS ONE style templates can be found at

https://journals.plos.org/plosone/s/file?id=wjVg/PLOSOne_formatting_sample_main_body.pdf and

<https://journals.plos.org/plosone/s/file?id=ba62/PLOSOne_formatting_sample_title_authors_affiliations.pdf>

**Response - The manuscript has been formatted according to PLOS ONE's style requirements.**

2. Please include additional information regarding the survey or questionnaire used in the study and ensure that you have provided sufficient details that others could replicate the analyses. For instance, if you developed a questionnaire as part of this study and it is not under a copyright more restrictive than CC-BY, please include a copy, in both the original language and English, as Supporting Information.  If the original language is written in non-Latin characters, for example Amharic, Chinese, or Korean, please use a file format that ensures these characters are visible.

**Response - The quality improvement implementation guide has been uploaded as a supplementary file (S1 Text). The individual training registration and evaluation form was used to collect data. This has been uploaded as a supplementary file (S2 Text)**

3. Please provide additional details regarding participant consent. In the ethics statement in the Methods and online submission information, please ensure that you have specified (1) whether consent was informed and (2) what type you obtained (for instance, written or verbal, and if verbal, how it was documented and witnessed). If your study included minors, state whether you obtained consent from parents or guardians. If the need for consent was waived by the ethics committee, please include this information.

**Response - Additional details regarding participant consent have been specified in the ethics statement section of the manuscript (Page 11, line 225-226).**

4.In your Data Availability statement, you have not specified where the minimal data set underlying the results described in your manuscript can be found. PLOS defines a study's minimal data set as the underlying data used to reach the conclusions drawn in the manuscript and any additional data required to replicate the reported study findings in their entirety. All PLOS journals require that the minimal data set be made fully available. For more information about our data policy, please see http://journals.plos.org/plosone/s/data-availability.

Upon re-submitting your revised manuscript, please upload your study’s minimal underlying data set as either Supporting Information files or to a stable, public repository and include the relevant URLs, DOIs, or accession numbers within your revised cover letter. For a list of acceptable repositories, please see http://journals.plos.org/plosone/s/data-availability#loc-recommended-repositories. Any potentially identifying patient information must be fully anonymized.

Important: If there are ethical or legal restrictions to sharing your data publicly, please explain these restrictions in detail. Please see our guidelines for more information on what we consider unacceptable restrictions to publicly sharing data: http://journals.plos.org/plosone/s/data-availability#loc-unacceptable-data-access-restrictions. Note that it is not acceptable for the authors to be the sole named individuals responsible for ensuring data access.

We will update your Data Availability statement to reflect the information you provide in your cover letter.

**Response - The minimal dataset underlying the results will be made available when the manuscript is accepted for publication.**

5. Your ethics statement should only appear in the Methods section of your manuscript. If your ethics statement is written in any section besides the Methods, please move it to the Methods section and delete it from any other section. Please ensure that your ethics statement is included in your manuscript, as the ethics statement entered into the online submission form will not be published alongside your manuscript.

**Response - The ethics statement has been included in the method section (Page11, line 222)**

6.Thank you for stating the following in the Financial Disclosure section:

[Funding was obtained from the Vetenskapsrådet (SE), the Laerdal Foundation for
Acute Medicine, Norway, and Einhorn Family Foundation, Sweden. AKC was the grantee of
Vetenskapsrådet (SE).MM was the grantee of Laerdal Foundation for Acute Medicine, Norway,
and Einhorn Family Foundation, Sweden. The funders had no role in study design, data collection
and analysis, decision to publish, or preparation of the manuscript.].

We note that one or more of the authors are employed by a commercial company: Golden Community, Lalitpur, Nepal

**Response - Golden Community is not a commercial company but a national research agency and is the sub-grantee of the Swedish Research Council fund for data collection of Nepal Perinatal Quality Improvement Project (NePeriQIP).**

1. Please provide an amended Funding Statement declaring this commercial affiliation, as well as a statement regarding the Role of Funders in your study. If the funding organization did not play a role in the study design, data collection and analysis, decision to publish, or preparation of the manuscript and only provided financial support in the form of authors' salaries and/or research materials, please review your statements relating to the author contributions, and ensure you have specifically and accurately indicated the role(s) that these authors had in your study. You can update author roles in the Author Contributions section of the online submission form.

Please also include the following statement within your amended Funding Statement.

“The funder provided support in the form of salaries for authors [insert relevant initials], but did not have any additional role in the study design, data collection and analysis, decision to publish, or preparation of the manuscript. The specific roles of these authors are articulated in the ‘author contributions’ section.”

**Response - The funder provided support in form of salaries for OB, but did not have any additional role in the study design, data collection and analysis**

If your commercial affiliation did play a role in your study, please state and explain this role within your updated Funding Statement.

**Response - none**

2. Please also provide an updated Competing Interests Statement declaring this commercial affiliation along with any other relevant declarations relating to employment, consultancy, patents, products in development, or marketed products, etc.

**Response - none**

Within your Competing Interests Statement, please confirm that this commercial affiliation does not alter your adherence to all PLOS ONE policies on sharing data and materials by including the following statement: "This does not alter our adherence to  PLOS ONE policies on sharing data and materials.” (as detailed online in our guide for authors [http://journals.plos.org/plosone/s/competing-interests](about:blank)) . If this adherence statement is not accurate and  there are restrictions on sharing of data and/or materials, please state these. Please note that we cannot proceed with consideration of your article until this information has been declared.

**Response - None of the authors have commercial affiliation**

Please include both an updated Funding Statement and Competing Interests Statement in your cover letter. We will change the online submission form on your behalf.

Please know it is PLOS ONE policy for corresponding authors to declare, on behalf of all authors, all potential competing interests for the purposes of transparency. PLOS defines a competing interest as anything that interferes with, or could reasonably be perceived as interfering with, the full and objective presentation, peer review, editorial decision-making, or publication of research or non-research articles submitted to one of the journals. Competing interests can be financial or non-financial, professional, or personal. Competing interests can arise in relationship to an organization or another person. Please follow this link to our website for more details on competing interests: [http://journals.plos.org/plosone/s/competing-interests](about:blank)

 7. Please note that in order to use the direct billing option the corresponding author must be affiliated with the chosen institute. Please either amend your manuscript to change the affiliation or corresponding author, or email us at plosone@plos.org with a request to remove this option.

[Note: HTML markup is below. Please do not edit.]

**Reviewers' comments:**

Reviewer's Responses to Questions

**Comments to the Author**

1. Is the manuscript technically sound, and do the data support the conclusions?

The manuscript must describe a technically sound piece of scientific research with data that supports the conclusions. Experiments must have been conducted rigorously, with appropriate controls, replication, and sample sizes. The conclusions must be drawn appropriately based on the data presented.

Reviewer #1: Yes

Reviewer #2: Yes

2. Has the statistical analysis been performed appropriately and rigorously?

Reviewer #1: Yes

Reviewer #2: Yes

3. Have the authors made all data underlying the findings in their manuscript fully available?

The [PLOS Data policy](http://www.plosone.org/static/policies.action#sharing) requires authors to make all data underlying the findings described in their manuscript fully available without restriction, with rare exception (please refer to the Data Availability Statement in the manuscript PDF file). The data should be provided as part of the manuscript or its supporting information, or deposited to a public repository. For example, in addition to summary statistics, the data points behind means, medians and variance measures should be available. If there are restrictions on publicly sharing data—e.g. participant privacy or use of data from a third party—those must be specified.

Reviewer #1: Yes

Reviewer #2: Yes

4. Is the manuscript presented in an intelligible fashion and written in standard English?

PLOS ONE does not copyedit accepted manuscripts, so the language in submitted articles must be clear, correct, and unambiguous. Any typographical or grammatical errors should be corrected at revision, so please note any specific errors here.

Reviewer #1: No

**Response - manuscript edited for language and grammatical errors.**

Reviewer #2: Yes

5. Review Comments to the Author

Please use the space provided to explain your answers to the questions above. You may also include additional comments for the author, including concerns about dual publication, research ethics, or publication ethics. (Please upload your review as an attachment if it exceeds 20,000 characters)

Reviewer #1: Abstract: the authors summarize the main research question and key findings succinctly.
Introduction: the authors do a sufficiently good job in setting the stage for the paper through several references quoted, leading finally to the research question. I would like to advice the authors to increase references for QI approaches and outcomes. The text in rows 87-89 requires revision. My suggestion would be to remove QI from the training approach and to note it as an implementation approach.

**Response - Edited as per the suggestions**

Intervention: Please clarify if the external mentors were employed by the public health sector or from the private sector

**Response - Specified (page 6, line 129)**

Results: Knowledge and skills scores were analyzed by hospital size, individual hospital, but the results were used to interpret the findings in a limited fashion. An interesting finding from this analysis noted skills deterioration was higher for high volume hospitals. This finding would benefit from understanding why that was so.

**Response - The suggested finding has been included in the result (Page 13, line 273)**

Discussion: I think the authors have done a really good job in setting up interventions and have a very interesting and valuable results from the data analyzed. Some of the results could be worth discussing. The ones that I would recommend for the authors to add their observations on the lack of statistical difference in retention of skills over time in high, medium and low volume hospitals and among the different providers under current position. Traditionally, we would have expected more opportunities for practice / implementation would have meant more retention of skills. That is not demonstrated by this study.

**Response - Observations regarding lack of statistical difference has been explained (page 19-20, line number 376 - 382)**

Overall write-up is clear and appealing and of value. There are a couple of minor typos that could benefit from a thorough copy-editing by the authors

**Response - Manuscript has been edited for language**

Reviewer #2: This is an interesting study evaluating the pre-post study design in 12 public hospitals of Nepal that is assessing the knowledge and skills of trainees on neonatal resuscitation. There is a clear need for increased knowledge and skills of neonatal resuscitation to help improve outcomes of neonates. This study evaluated a year+ long effort to train many providers on HBB and test their pre-post knowledge. It showed that the HBB with QI package is helpful in this setting to improve knowledge retention and hope future efforts will continue to build capacity there for this vulnerable population.

6. PLOS authors have the option to publish the peer review history of their article ([what does this mean?](https://journals.plos.org/plosone/s/editorial-and-peer-review-process#loc-peer-review-history)). If published, this will include your full peer review and any attached files.


If you choose “no”, your identity will remain anonymous but your review may still be made public.


**Do you want your identity to be public for this peer review?** For information about this choice, including consent withdrawal, please see our [Privacy Policy](https://www.plos.org/privacy-policy).

Reviewer #1: **Yes:** Neena Khadka

Reviewer #2: No

[NOTE: If reviewer comments were submitted as an attachment file, they will be attached to this email and accessible via the submission site. Please log into your account, locate the manuscript record, and check for the action link "View Attachments". If this link does not appear, there are no attachment files.]

While revising your submission, please upload your figure files to the Preflight Analysis and Conversion Engine (PACE) digital diagnostic tool, <https://pacev2.apexcovantage.com/>. PACE helps ensure that figures meet PLOS requirements. To use PACE, you must first register as a user. Registration is free. Then, login and navigate to the UPLOAD tab, where you will find detailed instructions on how to use the tool. If you encounter any issues or have any questions when using PACE, please email PLOS at [figures@plos.org](mailto:figures@plos.org). Please note that Supporting Information files do not need this step.

**Response - Figure file has been checked with PACE**

Yours Sincerely,

Dipak Raj Chaulagain, MPH (Corresponding author)

Uppsala Global Health Research on Implementation and Sustainability (UGHRIS),

Department of Women's and Children's Health, Uppsala University, Uppsala, Sweden

Email address: dipak.chaulagain@kbh.uu.se
