# Supplementary material for: Effect of a scaled-up quality improvement intervention on health workers’ competence on neonatal resuscitation in simulated settings in public hospitals: A pre-post study in Nepal
Source: PLoS One. 2021 Apr 29;16(4):e0250762. doi: 10.1371/journal.pone.0250762 (PMC8084235; doi:10.1371/journal.pone.0250762)
Supplement: Attachment — Submitted filename: Response to reviewers.rtf [file pone.0250762.s005.rtf]

Anne Lee Solevåg, 				                   01 April 2021
Academic Editor
PLOS ONE

Re: Comments addressed for the manuscript "Effect of a scaled-up quality improvement intervention on health workers' competence on neonatal resuscitation in simulated settings in public hospitals: a pre-post study in Nepal" (PONE-D-20-15505R1)

Dear Anne Lee,
Thank you very much for providing opportunity to revise our manuscript for the second time. We have attempted to address all the comments in our revised manuscript. Please find the responses (in bold) to each comment below;

Academic editor comments
1. In the ethics statement, if verbal consent was obtained, how it was documented and witnessed
Response - Additional details regarding participant consent have been specified in the ethics statement section of the manuscript (Page11, line 235-243). 
All participants were provided with verbal information on their participation in the study. The training registration and evaluation form (S2 Text), which was used to evaluate trainees' knowledge and skills in this study, was used for the documentation of their consent. The participants filled and signed the 'training registration and evaluation form ' after agreeing to participate in the study. The process of obtaining consent was completed in the presence of hospital management in the respective hospitals.  The signed individual training registration and evaluation forms have been securely stored at the central research office in Kathmandu. 

2. Reviewer comment: -I would like to advice the authors to increase references for QI approaches and outcomes
The references for QI approaches and outcomes have been increased (page 4,5, line 85-91)
 
Yours Sincerely,
Dipak Raj Chaulagain, MPH (Corresponding author)
Department of Women's and Children's Health, Uppsala University, Uppsala, Sweden
Email: dipak.chaulagain@kbh.uu.se
